# Strategies to control humidity sensitivity of azobenzene isomerisation kinetics in polymer thin films

**DOI:** 10.1038/s43246-024-00642-w

**Published:** 2024-10-02

**Authors:** Sami Vesamäki, Henning Meteling, Roshan Nasare, Antti Siiskonen, Jani Patrakka, Nelmary Roas-Escalona, Markus Linder, Matti Virkki, Arri Priimagi

**Affiliations:** 1 Faculty of Engineering and Natural Sciences, Tampere University, Tampere, Finland; 2 Department of Bioproducts and Biosystems, Aalto University, Espoo, Finland; 3 VTT Technical Research Centre of Finland Ltd, Oulu, Finland

**Keywords:** Polymers, Sensors and biosensors

## Abstract

Azobenzenes are versatile photoswitches that garner interest in applications ranging from photobiology to energy storage. Despite their great potential, transforming azobenzene-based discoveries and proof-of-concept demonstrations from the lab to the market is highly challenging. Herein we give an overview of a journey that started from a discovery of hydroxyazobenzene’s humidity sensitive isomerisation kinetics, developed into commercialization efforts of azobenzene-containing thin film sensors for optical monitoring of the relative humidity of air, and arrives to the present work aiming for better design of such sensors by understanding the different factors affecting the humidity sensitivity. Our concept is based on thermal isomerisation kinetics of tautomerizable azobenzenes in polymer matrices which, using pre-defined calibration curves, can be converted to relative humidity at known temperature. We present a small library of tautomerizable azobenzenes exhibiting humidity sensitive isomerisation kinetics in hygroscopic polymer films. We also investigate how water absorption properties of the polymer used, and the isomerisation kinetics are linked and how the azobenzene content in the thin film affects both properties. Based on our findings we propose simple strategies for further development of azobenzene-based optical humidity sensors.

## Abstract (web summary)

Many variables related to azobenzene-based humidity sensors remain. Here, a small library of tautomerisable azobenzenes is studied for their humidity dependent thermal isomerisation rate in polymer thin films.

## Introduction

Molecules that can be reversibly photoswitched between two states with different physical or chemical properties have received great interest in the modern photonics era^1,2^. When incorporated into materials, the molecular photoswitches provide photocontrol over plethora of properties, ranging from phase transition temperatures^3,4^ and adhesion^5^ to refractive index control^6,7^ and surface topography^8^. They have therefore found uses in fields such as chemical biology and photopharmacology^9–13^, solar energy storage^14–16^, electronics and logic systems^17,18^, and photo-controlled catalysis^19–21^. Among the most important properties of a photoswitch are, e.g., the switching wavelength(s), stability of the different states, solubility, and photostability. The specific requirements posed to a molecular photoswitch vary greatly depending on the application in question. For example, photocontrolled drug release benefits from switching with NIR-light and high switching quantum yield^22^, while dynamic holography benefits from photostability and fast switching between the states^23^. The need to modify photoswitching properties for each application creates great demand for photoswitch engineering.

Azobenzenes are arguably the most widely studied class of photoswitches and have garnered a lot of attraction as the photoswitches of choice for many applications^24–26^. One reason for this is the large molecular-level motions azobenzenes undergo upon isomerisation between the stable *trans* isomer and meta-stable *cis* isomer, making azobenzene-containing materials rather unique for, e.g., photoalignment, photopatterning^27,28^, and macroscopic photoactuation^29,30^. The key to azobenzenes’ popularity lies in the modifiability of their photoswitching properties by substitution on the phenyl rings. For example, near-quantitative photoisomerisation to both directions can be obtained with visible light via *ortho*-substitution^31,32^, and protonation enables pushing the switching wavelength all the way to near infrared^33^. Also the stability of *cis* isomer can be controlled over wide range, from milliseconds to years, with different substitution strategies^34,35^, including the use of heteroaromatic azo-compounds^36–38^.

Apart from the molecular structure, environmental factors such as temperature, polarity^39^, concentration^37,40^, and pressure^41^ play an important role in dictating the isomerisation kinetics of azobenzenes. A prominent example of this is offered by hydroxyazobenzenes that have orders-of-magnitude difference in thermal isomerisation rate in polar vs. non-polar solvents^34,36,42^. Such environmental dependence offers a dynamic range for isomerisation kinetics, i.e., systematic change in the *cis*-lifetime upon changes in some environmental variable. An example of this is the pH-dependent thermal isomerisation rate of hydroxy- and aminoazobenzenes in aqueous solutions^43^. If incorporated into solid matrices, the dynamic isomerisation behavior offers interesting possibilities for applications, such as gated photoresponsive systems and lifetime-based sensing^25^.

In the scope of sensing, azobenzenes have been utilized as colorimetric sensors^44–47^ that are based on changes in absorption spectrum upon binding of an analyte to an azobenzene-based probe. To the best of our knowledge, the first and so far the only example of sensing based on isomerisation kinetics of azobenzene is optical humidity sensing in hydroxyazobenzene-containing polymer thin films, demonstrated by Poutanen et al. ^48^. Alike in solution^42^, the proposed mechanism for the humidity sensitivity in the polymer films was hydrogen bond-assisted azo–hydrazone tautomerism. Yet many important questions remain: How does the sensitivity of the method depend on the chemical structure of the (hydroxy)azobenzene or the polymer matrix used? Is the phenomenon unique to hydroxyazobenzenes? Are the sensors reliable over a long enough period to be commercially viable? Answering these questions will be critical in putting the proposed sensing concept into use in practical device applications.

Herein, we delve into the above questions and present a small library of tautomerizable azobenzenes exhibiting humidity-dependent thermal isomerisation rate in polymer thin films. We also study the dependency of the sensing performance and the water absorption characteristics of the polymer matrix the azobenzenes are incorporated into and show that the azobenzene content profoundly influences both the hydrophilicity of the polymer system as well as the humidity dependence of the thermal isomerisation rate. We also summarize our recent efforts aiming towards realization of a working humidity sensing device and preparing it for commercialization, a path during which the need for additional fundamental research became clear. Our results showcase strategies to modify and optimize the thermal-isomerisation-based humidity sensing concept, paving the way towards in-depth understanding, better sensors, and in longer term, hopefully, commercial device applications.

## Results

### Sensing concept

**Fig. 1:**
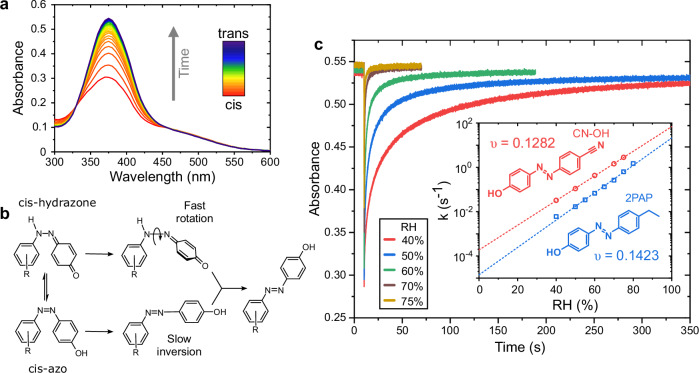
Humidity dependence of hydroxyazobenzene isomerisation kinetics. **a** Changes in absorption spectrum of CN-OH in P4VP film in response to UV illumination (385 nm, ~300 mW · cm^−2^, 100 ms pulse) and the subsequent *cis*–*trans* thermal relaxation. **b** Scheme of the main pathways for cis–trans isomerisation of tautomerizable hydroxyazobenzene. **c** Effect of relative humidity on the thermal isomerisation kinetics of CN-OH in P4VP thin film at 22 °C, measured at 365 nm. Inset: comparison between the humidity dependency of thermal isomerisation rates, *k*, of CN-OH and 2PAP in P4VP at 22 °C.

Determination of *cis* lifetime is straightforward for many azobenzene derivatives, thanks to the large differences in the absorption spectrum of the two isomers, as depicted by the diminished $\pi -{\pi }^{* }$ absorption band upon *trans*–*cis* isomerisation shown in Fig. 1a. The isomerisation kinetics can therefore be determined by measuring the absorption at a fixed wavelength or wavelength range after photoexcitation. In solution the thermal relaxation normally follows first-order kinetics but in solid phase the molecular movements are more restricted, and a stretched exponential model is better capable of describing the thermal isomerisation kinetics^49^ (see “Methods” for further details).

Figure 1b illustrates how tautomerization from the azo to hydrazone form activates the fast rotational pathway for the *cis–trans* isomerisation^50^. The proposed mechanism for the humidity-dependent isomerisation kinetics in polymer films is that introduction of hydrogen-bonding species such as water molecules to the system pushes the azo–hydrazone equilibrium towards the hydrazone form and thus increases the thermal isomerisation rate. Figure 1c shows this humidity dependence for two hydroxyazobenzenes, 4-(4-ethylphenylazo)phenol) (2PAP), the hydroxyazobenzene used in the original demonstration by Poutanen et al.^48^, and 4’-cyano-4-hydroxyazobenzene (CN-OH). The isomerisation rate constants at different relative humidities in poly(4-vinylpyridine) thin films are presented in the inset of Fig. 1c, indicating exponential dependency and almost 6 orders of magnitude dynamic range when extrapolated to 0–100% RH. The constant *υ* describes the degree of humidity sensitivity at a given temperature through the relation

1 $$k({{\rm{RH}}})={k}_{0}{{{\rm{e}}}}^{\upsilon \cdot {{\rm{RH}}}},$$

where ${k}_{0}$ is the rate constant at 0% RH. This indicates that 2PAP is slightly more sensitive to environmental humidity changes than CN-OH. However, it is also evident that thermal back relaxation of 2PAP is systematically slower than CN-OH, rendering it less practical from the perspective of device applications. For this reason, CN-OH was chosen over 2PAP as the workhorse molecule for the subsequent sensor development and the reference molecule for the present work.

### From sensing concept to practical sensing device

Soon after presenting the idea of humidity sensing based on azobenzene isomerisation kinetics^48^, we shifted our focus to turning the concept into a practical device. Our aims were both technical and commercial. On the technical side, the goal was to produce a device that can demonstrate the functionality of the technology and to develop sensor materials that enable measurements over wide range of conditions. The commercial side focused on finding an entry market for the new technology and gathering input from possible users to steer the technical development.

**Fig. 2:**
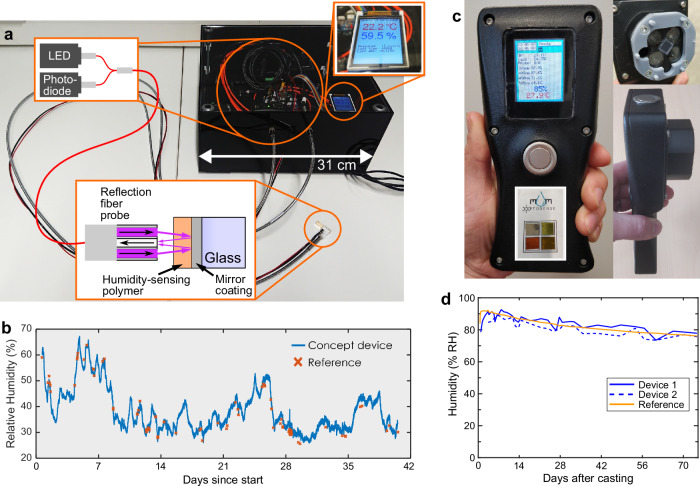
Proof of Concept devices for optical humidity sensing. **a** First-generation concept device with schematics of its working principle. **b** 6 weeks long experiment with the first-generation device continuously measuring ambient relative humidity. **c** Second-generation concept device capable of measuring with four wavelengths at the same time and a sticker type sensor-array with different hydroxyazobenzene-containing sensing materials. **d** 11 weeks long experiment with second-generation concept devices and sticker sensors measuring drying of concrete.

The first working concept device capable of automatically measuring the isomerisation kinetics of azobenzene in thin films and harnessing it for continuous monitoring of ambient humidity is shown in Fig. 2a. The device was rather bulky, but capable of accurate humidity measurement in ambient conditions at least over a period of 6 weeks (Fig. 2b). We proceeded towards commercial preparations via refinement of the measuring device and sensor materials to the requirements of potential users. The current state of the technology is a handheld concept device capable of measuring isomerisation kinetics at multiple wavelengths using separate light sources (Fig. 2c) and sensor materials suitable for 50–‍90% relative humidity range around room temperature. The design principle focuses on simple usage and portability and the use of multiple sensor materials aims to provide higher accuracy or the possibility of expanding the suitable relative humidity range for measurements. Each fabricated sensor is usable for hundreds if not thousands of cycles (Supplementary Figs. S34–S36), but also the ease and cost of fabrication makes the sensors easily replaceable. Another point of consideration is the safety of the materials. Many azobenzenes are considered non-toxic and azobenzene-based materials have been utilized, e.g., as cell culture platforms^51,52^. As for polymers we chose ones widely considered non-hazardous and opted to use solvents that are considered relatively safe, such as ethanol and ethyl acetate, when possible.

Even if the concept of using azobenzene isomerisation kinetics-based humidity sensing has now been shown to work, the technology has not yet reached commercial viability. The main technical reasons for this are inadequate accuracy of measurements and limited working range due to drastic changes in the measurement speed depending on the relative humidity. For a demonstration of the operationality of the sensor we looked for a high humidity environment where the relative humidity changes over time. For such purpose, freshly poured concrete casts proved very suitable, as the relative humidity needs the be monitored in the 100% to 85% RH range over prolonger time periods^53^. Drying concrete can also release volatile organic compounds^54^, making the environment quite harsh. We monitored the drying of fresh concrete casts with our sticker sensors and the 2nd-generation concept device for 11 weeks, as shown in Fig. 2d. The limited accuracy of our sensor is apparent compared to the industry standard reference device. The accuracy was within ±5% RH of the reference, but the difference between our two concept devices was at times rather large. Still, the sensors continued functioning reliably for the duration of the experiment despite the harsh conditions, showcasing their robustness, encouraging further development and optimization. As for the measurement speed, we consider *cis* lifetime in the range of 10 ms–10 s to be practical for humidity sensing purposes, allowing relatively fast measurements with simple optical detection schemes. The accuracy can be addressed by development of the measuring device and software, but the working range and measurement speed are determined by the sensor materials used. This demand for better understanding on how to control azobenzene isomerisation kinetics in polymer thin films inspired the work that is presented in the becoming sections.

### Material design principles

**Fig. 3:**
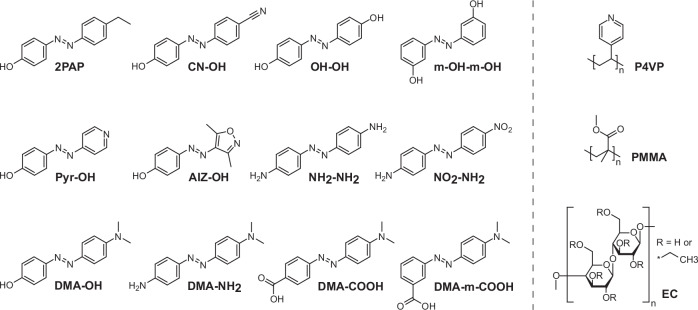
Chemical structures of the azobenzene molecules and polymer matrices used in this work. The selection of azobenzenes includes tautomerizable hydroxyazobenzenes, hydroxy-heteroazoarenes, and amino-azobanzenes, as well as nontautomerizable 3-3’-dihydroxyazobenzene (m-OH-m-OH). The polymers shown on the right were chosen to have a range of different water absorption characteristics.

The thermal *cis–trans* isomerisation rate of azobenzenes can be increased by appropriate modification of the azobenzene core, either by: (i) having strong electron donor–acceptor (D–A) configuration, typically in the 4, 4’ positions; (ii) having a structure capable of tautomerizing and partially breaking the N=N double bond; or (iii) combination of these features^55^. As already mentioned, hydroxyazobenzenes are capable for azo–hydrazone tautomerization, rendering the thermal isomerisation rate highly environment-sensitive, both in liquid phase^42,43^ and when incorporated into polymer matrix^48,56^. To verify the importance of tautomerization for the humidity sensitivity, the kinetics of 4-4’-dihydroxyazobenzene (OH-OH) and 3-3’-dihydroxyazobenzene (m-OH-m-OH), the latter being incapable of tautomerization, were compared. In terms of 4-hydroxyazobenzenes, we systematically varied the 4’-substituent’s electron affinity, providing a comparison between 2PAP, CN-OH, OH-OH, and 4-hydroxy-4’-dimethylamino-azobenzene (DMA-OH) (Fig. 3). Since the fastest isomerisation rates have been reported for heterocyclic azo-compounds^36,38^, we also included two heterocyclic hydroxyazobenzenes, 4-(Pyridin-4-yldiazenyl)phenol (Pyr-OH) and 4-[(4-hydroxyphenyl)diazenyl]-3,5-dimethylisoxazole (AIZ-OH), to our molecular library.

Hydroxyazobenzenes are not the only molecules capable of azo–hydrazone tautomerization. For example phenylazoindoles with tautomerizable structures exhibit very fast and highly environmentally sensitive isomerisation rates^38^. Also 4-aminoazobenzenes support tautomerization, yielding, e.g., pH-sensitivity of thermal isomerisation rate with a wide dynamic range^43^. With molecules 4,4’-Diaminoazobenzene (NH_2_-NH_2_), Disperse Orange 3 (NO_2_-NH_2_), and 4’-dimethylamino-4-aminoazobenzene (DMA-NH_2_) we set to study if 4-aminoazobenzenes exhibit humidity sensitivity like 4-hydroxyazobenzenes. Like with hydroxyazobenzenes, 4’-substituents with varying electron affinity were included. Finally, in the series DMA-OH, DMA-NH_2_ and 4’-carboxy-4-dimethyl-aminoazobenzene (DMA-COOH), we compared hydroxy- amino- and carboxylic acid as the 4-substituent, also varying the position of the carboxylic acid moiety (DMA-COOH vs. 3-Carboxy-4’-dimethylaminoazobenzene (DMA-m-COOH)). All studied azobenzene structures are shown in Fig. 3.

Another plausible approach to controlling the humidity sensitivity of the thermal isomerisation rate is the hygroscopicity of the polymer matrix used. Therefore, we compared three polymers (Fig. 3) with distinct water absorption properties. Poly(methyl methacrylate) (PMMA) was chosen as a weakly hygroscopic polymer^57^, poly-4-vinylpyridine (P4VP) as a hygroscopic polymer^58^, and ethyl cellulose as polymer with moderate hygroscopicity yet distinctly non-linear water absorption isotherm^59^. Another important selection characteristics for the chosen polymers was the presence of polar groups that ensures sufficient miscibility with hydroxyazobenzenes through hydrogen bonding, allowing for the use of desired molar ratio (from 1:8 up to 1:1) between the azobenzenes and the polymers’ repeating units without phase separation. In the absence of such polar groups and using, e.g., polystyrene, this turned out to be impossible.

### The effect of molecular structure

**Fig. 4:**
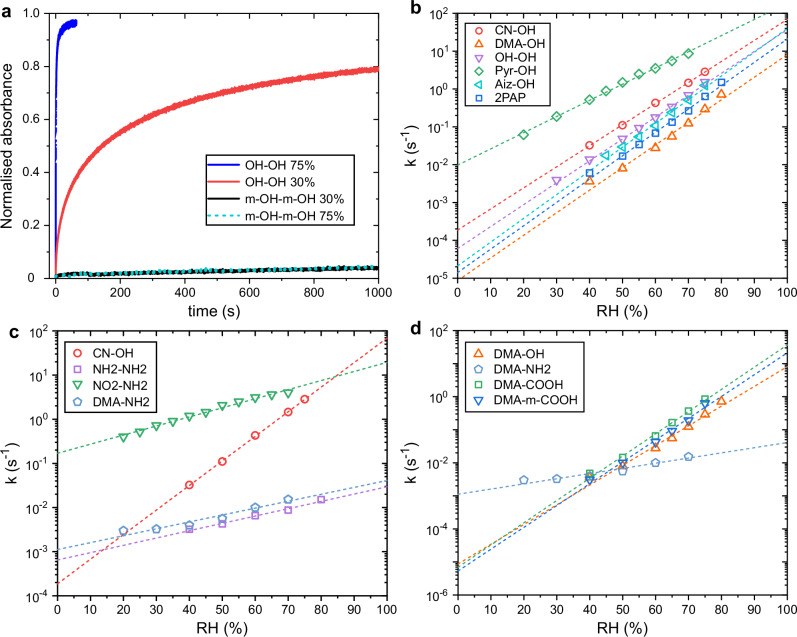
Humidity dependency of thermal isomerisation rate. **a** Thermal isomerisation kinetics of OH-OH and m-OH–m-OH at low (30 %) and high (75 %) relative humidities. Humidity-dependent thermal isomerisation rates, *k*, of (**b**) azo derivatives bearing 4-hydroxy group, (**c**) 4-aminoazobenzenes (CN-OH has been added to the graph for comparison purposes), and (**d**) 4-dimethylaminoazobenzenes with varying substituents. All molecules were studied in P4VP thin films using a molar ratio of 1:8. The wavelength at which absorbance was monitored, ${\lambda }_{\det }$, for m-OH-m-OH was 365 nm and for other azobenzenes is shown in Table 1.

First, to confirm that azo–hydrazone -tautomerization is required for the humidity sensitivity of hydroxyazobenzenes, we studied the isomerisation kinetics in the tautomerizable OH-OH and non-tautomerizable m-OH-m-OH at low and high humidities. As shown in Fig. 4a, the thermal isomerisation rate for m-OH-m-OH is extremely low (the *cis* lifetime of m-OH-m-OH in P4VP matrix at 22 °C is *ca*. 5 h), and no humidity dependency can be observed. In contrast OH-OH exhibits much faster, and highly humidity-dependent, thermal back isomerisation. This confirms that for hydroxyazobenzenes the ability to tautomerize to hydrazone form is crucial for humidity sensitive isomerisation kinetics.

As shown in Fig. 4b, all 4-hydroxyazobenzenes, irrespective of the para substituent, exhibit very strong humidity sensitivity in the thermal isomerisation rate with dynamic ranges of 4–6 orders of magnitude, being in principle all suitable for humidity sensing purposes. However, the para substitution or heteroaromaticity significantly affect the isomerisation rate at any given RH, for example at 50% RH DMA-OH has a *cis* lifetime of 120 s while for Pyr-OH it is only 670 ms. Having a strong electron donor in 4’-position makes the isomerisation slower and having an electron acceptor makes it faster. Regarding the heteroarenes, AIZ-OH behaves similarly as the other hydroxyazobenzenes while Pyr-OH is notably faster across the whole RH range with slightly weaker humidity sensitivity.

We next proceeded to study the humidity sensitivity of aminoazobenzenes (Fig. 4c). Albeit being somewhat humidity sensitive (dynamic range of two orders of magnitude or less), it is clear that the 4-hydroxyazobenzenes are superior compared to 4-aminoazobenzenes in terms of their humidity sensitivity. To illustrate this, the humidity dependent isomerisation kinetics of CN-OH is also presented in Fig. 4c. Like for hydroxyazobenzenes, the D–A configuration (NO_2_-NH_2_) significantly increases the rate of isomerisation but does not significantly affect the humidity sensitivity.

Figure 4d presents the humidity sensitivity of 4-dimethylaminoazobenzenes with hydroxy-, amino-, and carboxylic acid as para substituents. The DMA-NH_2_ and DMA-OH have comparable humidity sensitivity to other 4-amino- and 4-hydroxyazobenzenes, respectively, which demonstrates that 4-hydroxy substitution dominates over 4-amino substitution in boosting the humidity sensitivity. Interestingly, COOH substitution renders the isomerisation rate even more humidity sensitive than the 4-hydroxy substitution, with *υ* values of 0.155 and 0.152 for DMA-COOH and DMA-m-COOH, respectively. Furthermore, and unlike for hydroxy substitution, the humidity sensitivity of COOH-substituted dimethylaminoazobenzenes is not affected by the position of the carboxylic acid substituent, with DMA-COOH and DMA-m-COOH showing similar humidity dependencies. This is surprising since we had expected to see similar humidity sensitivity to other 4-aminoazobenzenes since the carboxylic acid itself is unable to facilitate tautomerization.

Table 1 gives a summary of the humidity sensitive kinetics of the molecules used in this study, with *cis* lifetime *τ* at 50% RH and 22 °C as well as in dry tetrahydrofuran (THF) giving an idea of the relative speed of isomerisation, sample-related constant *υ* describing the strength of humidity sensitivity, and the dynamic range giving the estimated limits of *τ* over the whole RH range at 22 °C. The wavelength of highest absorbance *λ*_max_ is also given for each sample. For absorbance spectra and humidity dependency data with error bars for each compound see Supplementary Note 1, Supplementary Figs. S37–S47.

**Table 1:** Humidity dependent isomerisation kinetics

Sample	*λ*_max_ [nm]	*λ*_det_ [nm]	*τ* (50% RH) [s]	*υ* [%^−1^]	Dynamic range of *τ* from 100% to 0% RH	*τ*(THF)
2PAP	360	365	59.5 ± 3.9	0.1423 ± 0.0024	47 ms–19.7 h	-^a^
CN-OH	374	365	9.1 ± 2.1	0.1282 ± 0.0017	15 ms–1.5 h	1.8 h
DMA-OH	416	405	125 ± 3	0.1375 ± 0.0036	124 ms–32.3 h	48.3 min
OH-OH	367	365	21.5 ± 1.5	0.1332 ± 0.0017	27 ms–4.6 h	2 h
Aiz-OH	346	365	34 ± 0.5	0.1445 ± 0.0021	25 ms–13.2 h	24.4 h
Pyr-OH	366	365	0.67 ± 0.14	0.0985 ± 0.0012	6 ms–103 s	48 s
NO_2_-NH_2_	457	430	0.48 ± 0.03	0.0474 ± 0.0011	53 ms–5.9 s	1.5 s
NH_2_-NH_2_	412	405	234 ± 25	0.0382 ± 0.0025	33 s–25.6 min	13.6 min
DMA-NH_2_	447	460	179.8 ± 10.8	0.0360 ± 0.0022	24 s–14.9 min	-^a^
DMA-COOH	442	430	69 ± 15	0.1549 ± 0.0027	27 ms–40.3 h	28.6 min
DMA-m-COOH	427	430	100 ± 8	0.1519 ± 0.0038	48 ms–52.4 h	22 min

*λ*_max_ is the wavelength of the highest absorption peak and *λ*_*det*_ is the monitoring wavelength for kinetics measurements. *τ*(50% RH) is the *cis*-lifetime at 50% relative humidity at 22 °C in seconds, *υ* is a sample related constant describing the strength of humidity sensitivity by relation $k\left({{\rm{RH}}}\right)={k}_{0}{{{\rm{e}}}}^{\upsilon \cdot {{\rm{RH}}}}$, Dynamic range is the extrapolated limits of *τ* at 100% and 0% RH at 22 °C in intuitive time units, and *τ*(THF) is *cis* lifetime in dry THF.

^a^*cis* lifetime was not determined in THF.

To further explain some of the trends observed in *cis* lifetime and humidity sensitivity the inversion and rotational isomerisation via hydrazone pathways were studied computationally by density functional theory (DFT) calculations in THF. The energies of the *cis*-azo and *cis*-hydrazone tautomers of 4-hydroxy- and 4-aminoazobenzenes without carboxylic acid substituents were calculated and major differences in their behavior were observed. For 4-hydroxyazobenzenes the *cis*-hydrazone tautomers have only slightly higher energy (1–2.5 kcal · mol^−1^) than the corresponding *cis*-azo tautomers indicating easy tautomerization even at room temperature. In comparison, 4-aminoazobenzenes have much higher energy gap between the *cis*-tautomers (9.9–13.2 kcal · mol^−1^), making tautomerization much less likely.

**Fig. 5:**
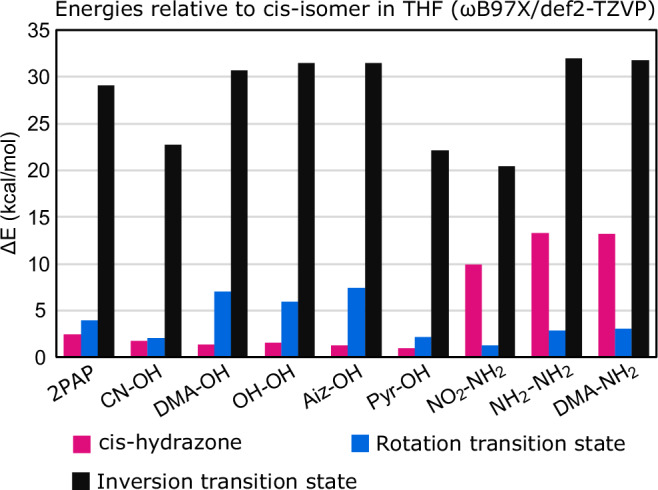
Calculated energies for different isomerisation pathways. Energies, Δ*E*, of *cis*-hydrazone and isomerisation transition states via different pathways compared to *cis*-azobenzene in THF, calculated using DFT.

The transition state energies of the rotation around the N-N bond for the hydrazone tautomers were also calculated, as we assumed fast isomerisation through this pathway. The transition states are only 1.3–7.4 kcal · mol^−1^ higher in energy than the corresponding *cis*-hydrazones for 4-hydroxy- and 4-aminoazobenzenes, which supports our assumption. Furthermore, the hydrazone transition state energies agree with the observed isomerisation rates, with low energy corresponding to generally faster isomerisation. Finally, the transition state energies for *cis-trans* isomerisation via inversion were calculated and based on the results the studied azobenzenes can be divided into two groups, with CN-OH, Pyr-OH and NO_2_-NH_2_ having a significantly lower inversion energy (20.7–22.7 kcal · mol^−1^) compared to the others (29.1–32.0 kcal · mol^−1^). This also agrees quite well with our observations since low inversion energy indicates faster thermal isomerisation even in absence of water. Together these results imply that the humidity sensitivity is governed by the affinity to tautomerize to hydrazone, while the general rate of isomerisation is governed by the transition state energies. The tautomerization and transition states for which the calculations were done are illustrated in Fig. 1b for 4-hydroxyazobenzene and the calculated energies for each azobenzene are shown in Fig. 5, as well as listed in Supplementary Table S3.

Based on our experimental observations and computational studies we propose that the humidity sensitivity of *cis* lifetime is not simply due to hydrogen bonding but rather is caused by proton transfer to one of the nitrogens forming the azo bond which pushes the equilibrium towards hydrazone, allowing for fast rotational isomerisation. This proton transfer is in turn promoted by the presence of water. This would explain why also carboxylic acid in the *meta* position is able to introduce high humidity sensitivity to azobenzene with tautomerizable DMA-substituent, even though the hydrogen donor and tautomerizable structure are not the same.

### The effect of polymer matrix

The azobenzene molecules embedded in a polymer film are not directly in contact with air. Thus, the relative humidity of air and the *cis* lifetime are bound together through the water absorption properties of the polymer film. The polymer first used to demonstrate systematic humidity dependency of isomerisation rate, P4VP, is a logical choice as it is (i) hygroscopic^58^ and therefore water content in the polymer is expected to rise rapidly with increasing air humidity and (ii) it is able to form supramolecular complexes with molecules containing hydrogen-bond-donating groups such as hydroxyazobenzenes, which supports high azobenzene loading without phase separation^60,61^. For our interests it is important to compare polymers with distinctly different water absorption characteristics to see how they translate to humidity sensitivity of thermal isomerisation.

We chose PMMA as a polymer with low water absorption and quite linear water absorption isotherm^57^ and ethyl cellulose (EC) as a polymer known to be hygroscopic, yet with distinctly non-linear absorption isotherm^59^. The water absorption isotherms of spin coated thin films of the three polymers were determined using a quartz crystal microbalance with dissipation (QCM-D) at 23 °C. The humidity sensitivity of CN-OH isomerisation kinetics was then investigated in these polymers using 1:8 molar ratio between CN-OH and polymer repeat units, which equates to 21−22 wt% of CN-OH in P4VP and PMMA, and 10.7 wt% in EC. The CN-OH content was chosen so that CN-OH should not have too great of an impact on the water absorption properties of the films and to reduce the interactions between the azobenzene molecules, but to also keep the absorbance levels of the azobenzene high enough for accurate measurements.

**Fig. 6:**
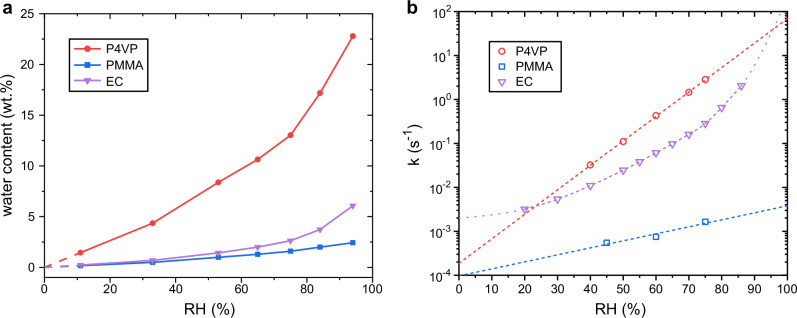
Water absorption isotherms for pure polymer films and humidity dependency of isomerisation rate in different polymers. **a** Water absorption isotherms of pure polymer films determined with QCM-D, expressed as wt% of water in the films (${{\rm{water\; wt}}}. \% =\frac{{m}_{{{\rm{water}}}}}{{m}_{{{\rm{film}}}}}\cdot 100 \%$) (**b**) Humidity dependency of isomerisation rate *k* of CN-OH in different polymer films.

The absorption isotherms for the polymers are shown in Fig. 6a. We found that at 94% RH PMMA absorbs 2 wt% and EC 6 wt% of water, in relatively good agreement with literature values of 2 wt%^57^ and 8 wt%^59^, respectively. The shapes of the isotherms are also in agreement with earlier findings with the isotherm for PMMA being almost linear and EC showing clearly superlinear behavior. For P4VP the amount of absorbed water at 94% RH was remarkably high, almost 23 wt%. The shape of the isotherm is quite linear up to 75% RH, from where the absorption accelerates a little. The humidity dependency of CN-OH isomerisation rate in the studied polymers is shown in Fig. 6b. Strong correlation was found between the humidity sensitivity and water absorption isotherms of pure polymers. The isomerisation rate in PMMA is much lower than in P4VP and EC and the humidity dependency is much weaker, as would be expected since there is less water in the film. The humidity dependency in EC has a similar shape to the water absorption isotherm of the polymer, with superlinear water absorption translating to faster than a simple exponential change in isomerisation rate. The isomerisation rates in EC are not much slower than in P4VP even though the amounts of water absorbed by the polymers are vastly different. A possible explanation for this is the presence of hydroxy groups in the EC itself. This could increase the rate regardless of water content as discussed in the case of azobenzene content.

For P4VP the used RH range has a linear absorption isotherm, which nicely translates to exponential isomerisation rate dependency. Unfortunately, our experiments were practically limited to relative humidities below 80%. Hence, we were unable to properly characterize the isomerisation kinetics in the superlinear regime of P4VP’s absorption isotherm, which could have given further confirmation on the relationship between the kinetics of azobenzene and water absorption characteristics of the polymer. In fact, the simple exponential humidity dependency observed with P4VP films might just be an artifact caused by too narrow RH range in the experiments. Upon close inspection, deviations from the exponential trend that suggest a faster dependency can be observed in samples containing DMA-NH_2_ and DMA-OH (Fig. 4) as well as 2PAP (Fig. 2). This means that the linear fits shown in the figures should only be used as a basis for comparison between the different molecules’ performance, rather than as projected performance at the extremes. Note, however, that as evident from Fig. 2d in monitoring the drying of concrete, the sensing concept is shown to work even at >90 RH% even if the sensing accuracy should be further improved.

### Effect of azobenzene content

The phenol–pyridine hydrogen bonding between hydroxyazobenzenes and P4VP enable fabrication of temporally stable films with high optical quality and very high (up to equimolar ratio) azobenzene content^61^. Since the investigated azobenzenes are hydrophobic it is expected that high azobenzene content significantly decreases water absorption in the film. We studied this effect in P4VP films by systematically increasing the azobenzene content. Increasing the amount of azobenzene also makes interaction between azobenzene molecules more probable and can thus change the isomerisation kinetics^62^. We measured absorption isotherms and humidity sensitivity of isomerisation rate for three CN-OH/P4VP complexes with different CN-OH contents. The chosen complexes have 1:1, 1:4 and 1:8 molar ratio of CN-OH to P4VP monomeric units, equating to 67, 35 and 21 wt% of CN-OH, respectively.

**Fig. 7:**
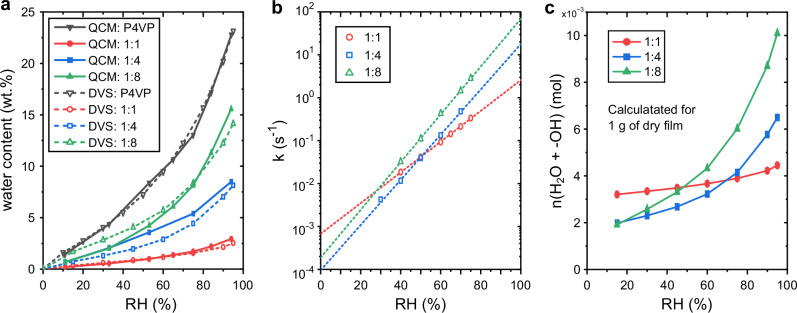
Effect of azobenzene content on water absorption and humidity sensitivity of thermal isomerisation rate. **a** Water absorption isotherms of P4VP films with varying CN-OH content, measured with QCM-D and DVS, expressed as wt% of water in the films (water wt.% = $\frac{{m}_{{{\rm{water}}}}}{{m}_{{{\rm{film}}}}} \cdot 100$%). **b** Humidity dependency of thermal isomerisation rate *k* in films with varying CN-OH content. **c** Calculation of total amount of water molecules and hydroxy groups, *n*(H_2_O + −OH), from azobenzenes in polymer films with different azobenzene content at different relative humidities. The amount of water was calculated from the data shown in Fig. 7a.

Like for pure polymers the water absorption isotherms were measured with QCM-D, but additionally verified via dynamic vapor sorption (DVS) measurements. Isotherms from both methods are shown in Fig. 7a. With QCM-D the absorbed water in the films at 94% RH and 23 °C for pure P4VP and the 1:8, 1:4 and 1:1 complexes were 22.7, 15.6, 8.5 and 2.9 wt%, respectively, showing how drastically the addition of less hygroscopic azobenzene reduces water absorption in the film. DVS results agree well with the QCM-D results with less than 2 wt% difference in absorbed water at the highest humidity for all samples. The dissipation measured by the QCM-D instrument gives information on the changes in mechanical properties of the film. The changes in dissipation measured in the films under study were not large (Supplementary Figs. S28–S33), but it increased slightly at highest humidities, meaning that the films become less rigid which in itself might also affect the isomerisation kinetics.

The humidity sensitivity of CN-OH’s isomerisation rate decreases with the increase in azobenzene content, as shown in Fig. 7b, which is expected since the amount of water absorbed by the film decreases. The *υ*-values, describing the humidity sensitivity, for the 1:1, 1:4 and 1:8 samples are 0.0825, 0.1213 and 0.1282, respectively. The difference between 1:4 and 1:8 samples is not large but clearly measurable, and the difference becomes more pronounced with the 1:1 sample. This can be rationalized by considering that less water is absorbed by the films with high azobenzene content, leading to lesser interactions between water and azobenzene molecules and thus reducing its effect on the isomerisation rate. Another noteworthy feature is that the 1:1 complex has faster isomerisation rate at low humidities than the samples with lower azobenzene content. This might be due to intermolecular interactions between the azobenzene molecules due to closer packing within the polymer film.

The effect of azobenzene content on the isomerisation kinetics in Fig. 7b is clear but the exact mechanism needs further analysis. We give a possible qualitative explanation with an assumption that each water molecule and hydroxy group in the azobenzenes affect the isomerisation kinetics in the same way. The number of hydroxy groups stays the same regardless of the water content in the film. Thus, high azobenzene content increases the isomerisation rate at low humidities. On the other hand, high azobenzene content decreases the amount of water absorbed by the film and thus diminishes its effect on the isomerisation rate. In Fig. 7c the total amount of water molecules and hydroxy groups from azobenzene molecules, n(H2O + -OH), in complexes with different azobenzene content at different relative humidities has been calculated from the absorption isotherms in Fig. 7a. This means that at low humidities the high-azobenzene-content films have more hydroxy groups that might assist tautomerization, but at high humidities, due to the reduced water absorption, the total amount of the tautomerization-assisting species is lower than in low-azobenzene-content films. This qualitatively explains the observed changes in isomerisation rate, offering one potential contributing factor to the complex dependencies observed. Yet more detailed analysis would be needed for quantitative understanding.

## Discussion

We have investigated humidity-dependent isomerisation kinetics of azobenzenes in polymer thin films and present three simple strategies to control it: the choice of azobenzene, the choice of polymer matrix, and the azobenzene content in the polymer matrix. Based on detailed structure–property studies and DFT calculations we propose that rotational isomerisation due to azo–hydrazone -tautomerization, which in turn is promoted by the presence of water, is the key to the humidity-dependent isomerisation kinetics. The role of the polymer matrix is to control the amount of water that can interact with the azobenzene. The amount of azobenzene in the polymer changes the water absorption characteristics of the film while also affecting the intermolecular interactions between the azobenzene molecules that can also have an effect on the isomerisation kinetics. Both the choice of azobenzene and the polymer matrix are critical for dictating the humidity sensitivity of the isomerisation kinetics. The azobenzene content, in turn, can be used to fine tune the sensitivity and absorption characteristics of the sensing layer. Better understanding of the underlying mechanisms of humidity sensitivity of the isomerisation kinetics will however help with the design of new sensor materials.

The azobenzene structure is the core of the humidity sensing concept. From the results presented in this work certain aspects affecting the humidity sensitivity of thermal isomerisation can be observed. First, to have humidity sensitivity in the first place a tautomerizable molecular structure is needed, as demonstrated by the lack of humidity sensitivity in m-OH-m-OH. While 4-aminoazobenzenes exhibit some humidity sensitivity it is much weaker than for 4-hydroxyazobenzenes or azobenzene carboxylic acids with dimethylamino substitution. By comparing the experimental and computational results, one can conclude that the highest humidity sensitivity is observed for compounds which can easily tautomerize to hydrazones, but do not easily isomerise via inversion, therefore preferring rotational isomerisation via hydrazone. It is also possible to control the isomerisation rate irrespective of humidity by D–A substitution and heteroaromaticity while maintaining the humidity sensitivity.

The choice of polymer matrix is as important as the azobenzene choice when considering the sensing performance since the polymer mediates the interaction between the azobenzene and the environment. Two main aspects to consider when choosing a polymer for senor materials are the water absorption characteristics and interactions with the azobenzene. The water absorption characteristics of the polymer directly affects the dynamic range of isomerisation rate by dictating how much water there is to interact with the azobenzenes. Our results suggest that with linear water absorption isotherm, the dependency between thermal isomerisation rate and relative humidity is exponential, while superlinear isotherm leads to deviations from this simple dependency.

The choice of azobenzene content in the film allows for fine tuning of the sensing performance. However, the challenge with this strategy becomes a balancing act between the desired sensitivity and stability of the films, as phase separation becomes more prominent when the amount of azobenzene is increased. This is clearly seen in films imaged with polarized optical microscope (Supplementary Fig. S15), with 1:8 CN-OH/P4VP showing practically no phase separation a year after fabrication and 1:1 film showing significant aggregation of CN-OH after only 4 months. Nevertheless, specific non-covalent interactions such as phenol–pyridine hydrogen bonding between the azobenzenes and the polymer matrix enable to significantly increase the azobenzene concentration, allowing the use of thinner sensing layers with shorter equilibration time.

In summary, there is no single material that ticks all the boxes for humidity sensing purposes. Rather, when designing a new sensor material, one must consider what is the most important characteristic it must fulfill (accuracy, speed, humidity range, longevity, …). The choice of azobenzene dictates the speed, accuracy and working range of the sensor. For accurate sensors 4-hydroxyazobenzenes and tautomerizable azobenzene carboxylic acids are good choices, but they yield slow measurements at low relative humidities. For fast measurements over wide RH range, at the expense of accuracy, 4-aminoazobenzenes with strong D–A configuration would be a decent choice, although they might be more prone to phase separation due to strong dipolar interactions between the azobenzene molecules. The polymer material is also a crucial when considering the speed and accuracy of the. For accurate sensors a polymer that absorbs significant amounts of water and preferably with linear water absorption isotherm, is optimal. P4VP fulfills these requirements rather well. Materials that absorb even more water could still allow for wider working ranges for fast measurements, but one must keep in mind that introduction of large amounts of water might have unforeseen effects on the thermal isomerisation kinetics. Also, absorption and desorption of water should be fast in an ideal sensor material to avoid hysteresis. For longevity of the sensors the choice of azobenzene and polymer material should be made so that the two materials bind together via relatively strong intermolecular forces such as hydrogen bonds. With the chosen materials the sensing performance can then be tweaked by the azobenzene content, with low content giving more accuracy in exchange of speed at low humidities and vice versa with high content. The azobenzene content also plays a significant role in the longevity, since large amount of azobenzene can lead to faster phase separation through stronger interactions between azobenzene molecules. The strategies we have presented here are simple yet crucial to understand for effectively transitioning our sensing technology into practical applications.

## Methods

### Materials

4-cyano-4’-hydroxyazobenzene (CN-OH, >95% purity) was bought from OTAVA chemicals, 4-hydroxy-4’-dimethylamino-azobenzene (DMA-OH, >98.0% purity), 4’-carboxy-4-dimethyl-aminoazobenzene (DMA-COOH, >97.0% purity), 4,4’-dihydroxyazobenzene (OH-OH) and 4’-dimethylamino-4-aminoazobenzene (DMA-NH_2_) from TCI, Disperse Orange 3 (NO_2_-NH_2_, dye content 90%) from Sigma-Aldrich, and 4,4’-Diaminoazobenzene (NH_2_-NH_2_, 95% purity) from Alfa Aesar. All compounds were used as received without further purification.

Synthesis of 4-[(4-hydroxyphenyl)diazenyl]-3,5-dimethylisoxazole (AIZ-OH) was adapted from a published route^63^ (Supplementary Methods, Synthesis 1), 3-3’-dihydroxyazobenzene was synthesized by homocoupling of TBDMS-protected 3-Hydroxyaniline (Supplementary Methods, Synthesis 2), 3-Carboxy-4’-dimethylaminoazobenzene (DMA-m-COOH)^64^ (Supplementary Methods, Synthesis 3), 4-(Pyridin-4-yldiazenyl)-phenol (Pyr-OH)^65^ (Supplementary Methods, Synthesis 4), and 4-(4-Ethylphenylazo)phenol (2PAP)^66^ (Supplementary Methods, Synthesis 5) were synthesized according to published procedures. The structures and purity of the synthesized compounds were verified by NMR spectroscopy (JNM-ECZR 500, JEOL) and mass spectrometry (JMS-T100LP AccuTOF LC-plus 4G, JEOL) (Supplementary Figs. S1–‍S14).

P4VP (avg. M_w_ = 60,000 g · mol^−1^), polystyrene (avg M_w_ = 350,000 g·mol^−1^), PMMA (avg M_w_ = 280,000 g·mol^−1^) and ethyl cellulose (48.0–49.5% (w/w) ethoxyl basis) were bought from Sigma-Aldrich and used as received. Ethanol (Etax A, ≥96.1 V-%), ethyl acetate (≥99.5%), and dimethylformamide (DMF, ≥99.8%) were purchased form Anora, Honeywell, and Sigma-Aldrich, respectively.

### Film fabrication

Thin film samples were prepared by first dissolving polymer and azobenzene in either ethanol, ethyl acetate, DMF, a mixture of ethanol and DMF, or a mixture of ethanol and ethyl acetate in desired molar ratios (Supplementary Table S1 for details). For Ethyl Cellulose (EC) films the ratio was fixed to have one azo molecule per 8 hydroxy groups in the polymer. Films were then spin coated (WS-650MZ-23NPPB, Laurell Technologies) on clean glass substrates at 1000 rpm for 40 s and left to dry on a 60 °C hot plate for 5–10 min. Films fabricated from pure DMF solution were kept under vacuum at 40 °C overnight to evaporated excess solvent. Film thicknesses were measured for a few samples with atomic force microscope (Dimension Icon, Bruker) and were found to be around 150–300 nm (Supplementary Figs. S24–S27).

### Absorption and thermal isomerisation measurements

The absorption spectra of the films were measured in ambient conditions right after fabrication (Cary 60 UV–vis, Agilent Technologies). The kinetics of the thermal back relaxation was measured inside a humidity-controlled chamber (LTS420 with RH95 relative humidity controller, Linkam Scientific Instruments) using a deuterium-halogen light source (DH-2000 BAL, Ocean Optics) for monitoring light and a fiber-optic spectrometer (AvaSpec-ULS2048L, Avantes) for detection. Neutral density filters (Thorlabs) were used as needed to avoid detector saturation and narrow band pass filters (Thorlabs) to limit monitoring wavelengths close to the absorption maximum of the *π*–π^*^ band. Photoexcitation was done with short 100 ms pulses from led light source with selectable wavelength (Lumen 1600-LED, Prior Scientific) using wavelengths slightly longer than the absorption maximum of the *π*–π^*^ band (Supplementary Table S1). The thermal isomerisation typically follows first-order kinetics in solution, but in solid phase the Kohlrausch−Williams−Watts function, which is a stretched exponential function, is better able to describe the kinetics with a single rate constant and a stretching term:

2 $$A\left(t\right)=\left({A}_{0}-{A}_{\infty }\right){{{\rm{e}}}}^{{\left(-{kt}\right)}^{\beta }}+{A}_{\infty }$$

where *A*_0_ is absorbance right after illumination, *A*_∞_ is absorbance of the fully relaxed state, *k* is the isomerisation rate constant and *β* is the exponential stretching term^49^.

Lifetimes for most of the azobenzenes were also measured in THF and lifetime for CN-OH was measured also THF with added water. Solution measurements were done with Cary 60 UV–vis (Agilent Technologies) spectrophotometer and Lumen 1600-LED (Prior Scientific) light source for the photoexcitation. For further details and results see Supplementary Methods, Supplementary Table S2 and Supplementary Figs. S16–S23.

### Computational studies

The molecular geometry optimizations and energy calculations were performed at the wB97X/def2-TZVP level of theory using ORCA 5.0.4. Frequency calculations were performed for all optimized geometries at the same level of theory to ensure that the obtained geometries were true minimum energy geometries (i.e., no imaginary frequencies). The conductor-like polarizable continuum method (CPCM) was used to account for solvent effects.

### Water absorption isotherms

Water absorption isotherms were measured using quartz crystal microbalance with dissipation (QCM-D) (Q-Sense E4, Biolin Scientific) equipped with a humidity module (QHM 401, Biolin Scientific). Films for QCM-D were directly deposited on gold coated sensors (QSX 301, Biolin Scientific), using a spin coater (WS-650SX-6NPP/LITE, Laurell Technologies). QCM-D measurements were done at 23 °C. Humidity inside the chamber was controlled with flow of saturated salt solution, with a water vapor permeable membrane separating the solution from the sensor. More information on the used salt solutions and QCM-D data analysis are found in Supplementary Methods, Supplementary Table S4 and Supplementary Figs. S28–S33. Absorption isotherms were also measured with dynamic vapor sorption technique (DVS Adventure, Surface Measurement Systems) to verify accuracy. The instrument requires milligrams of the film for accurate measurements, which meant that multiple films had to be scraped off the glass substrate to fit enough of it in the sample holder. In DVS experiments the relative humidity was increased from 0 to 95% in 5–15% steps and each humidity was held for 12 h. DVS measurements were done at 20 °C for pure P4VP and at 23 °C for other samples.

## Supplementary information

SI file

## Data Availability

The raw experimental data and analyzed data collected and used in this work, as well as custom MATLAB-code used for calculating the rate constants of thermal *cis-trans* isomerisation have been archived in Zenodo^67^.
