# Supplementary material for: Strategies to control humidity sensitivity of azobenzene isomerisation kinetics in polymer thin films
Source: Commun Mater. 2024 Oct 2;5(1):209. doi: 10.1038/s43246-024-00642-w (PMC11446815; doi:10.1038/s43246-024-00642-w)
Supplement: Supplementary file 1 — SI file [file 43246_2024_642_MOESM1_ESM.pdf]

Supplementary Information

Strategies to control humidity sensitivity of azobenzene isomerisation kinetics in polymer thin films

Sami Vesamäki[1], Henning Meteling[1], Roshan Nasare[1], Antti Siiskonen[1] , Jani Patrakka[1], Nelmary Roas Escalona[2], Markus Linder[2], Matti Virkki[3], and Arri Priimagi[1*]

[1] Faculty of Engineering and Natural Sciences, Tampere University, Korkeakoulunkatu 3, FI-33720 Tampere, Finland

[2] Department of Bioproducts and Biosystems, School of Chemical Engineering, Aalto University, FI-00076 Aalto, Finland

[3] VTT Technical Research Centre of Finland Ltd, Kaitoväylä 1, PL 1100, FI-90571 Oulu, Finland

* Corresponding Author E-Mail: arri.priimagi@tuni.fi

Table of Contents

Supplementary Methods 2

Azobenzene Synthesis 2

Synthesis 1: (E)-4-((4-hydroxyphenyl)diazenyl)-3,5-dimethylisoxazole (AIZ-OH) 2

Synthesis 2: 3,3’-Dihydroxyazobenzene (m-OH-m-OH) 4

Synthesis 3: 3'-Carboxy-4-dimethylaminoazobenzene (DMA-m-COOH) 7

Synthesis 4: 4-(Pyridin-4-yldiazenyl)phenol (Pyr-OH) 9

Synthesis 5: 4-(4-Ethylphenylazo)phenol (2PAP) 10

Thin film sample preparation and isomerisation wavelengths 11

Polarized optical microscopy images of films 12

Lifetime of studied azo-compounds in solution 13

Computational studies 18

AFM imaging of thin films 19

QCM measurements 21

Salt solutions 21

Data analysis 21

Measured responses 22

Repeated illumination test 25

Supplementary Note 1: Absorption spectra and humidity dependency of thermal isomerisation rate of individual azobenzenes in poly (4-vinyl)pyridine 28

Supplementary References 32

Supplementary Methods

Azobenzene Synthesis

Methodology (MS and NMR)

Accurate mass experiments were performed with the JEOL JMS-T100LP AccuTOF LC-plus 4G Time-of-Flight mass spectrometer using electrospray ionization. Nuclear magnetic resonance spectra (NMR) were measured with a 500 MHz JEOL ECZR 500 (125 MHz for ^{13}C) at 25 °C and processed with the JEOL Delta NMR software version 6.0.0 (Windows). Chemical shifts are given in ppm and are referenced to solvent signals (e.g. $CHCl_3$: δ = 7.26 ppm (^1H), 77.16 ppm (^{13}C)). Multiplicities are abbreviated as follows: singlet (s), doublet (d), doublet of doublets (dd), triplet (t), quartet (q), pentet (p), and multiplet (m). Coupling constants (J) are given in Hz.

Synthesis 1: (E)-4-((4-hydroxyphenyl)diazenyl)-3,5-dimethylisoxazole (AIZ-OH)

Synthesis of AIZ-OH adapted from L. Kortekaas et al.[1]

Synthesis of 3-(2-(4-hydroxyphenyl)hydrazineylidene)pentane-2,4-dione

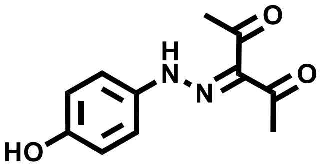

4-Aminophenol (0.966 g, 8.85 mmol, 1.0 eq) was dissolved in conc. acetic acid (17 M, 15 mL) and conc. hydrochloric acid (12 M, 2 mL) and cooled to 0 °C. $NaNO_2$ (0.770 g, 11.15 mmol, 1.3 eq) was dissolved in a minimum amount of Milli Q water and added dropwise to the 4-Aminophenol. The solution was stirred 60 minutes while keeping the temperature at 0 °C. Pentane-2,4-dione (1.175 g, 28.9 mmol, 1.3 eq) and NaOAc (2.37 g, 28.9 mmol, 3.3 eq) were suspended in a mixture of H_2O (5.5 mL) and EtOH (9 mL). The diazonium salt was then added dropwise to the suspension and the resulting mixture was stirred at room temperature overnight. The precipitate was filtered off, washed with Milli Q and dried in vacuum to yield the desired compound.

Molecular formula: $C_{11}H_{12}N_2O_3$

Yield: 95 % (1.86 g, 8.45 mmol)

^{1}H-NMR (500 MHz, DMSO-D6) δ = 9.71 (s, 1H), 7.41 (d, J = 6.3 Hz, 2H), 6.81 (d, J = 8.6 Hz, 2H), 2.40 (s, 6H)

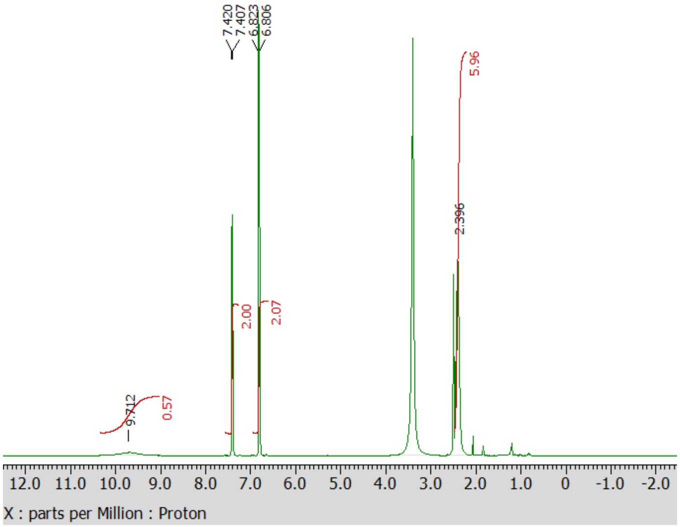

Supplementary Figure S1: ^{1}H-NMR spectrum of 3-(2-(4-hydroxyphenyl)hydrazineylidene)pentane-2,4-dione.

Synthesis of (E)-4-((4-hydroxyphenyl)diazenyl)-3,5-dimethylisoxazole (AIZ-OH)

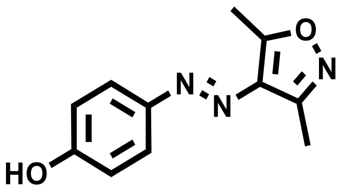

The synthesized arylhydrazonopentane-2,4-dione (1.823 g, 8.28 mmol, 1.0 eq) was dissolved in EtOH (35 mL) and AcOH (2.5 mL). $H_2NOH*HCl$ (0.860 g, 12.37 mmol, 1.5 eq) was added. The mixture was refluxed at 85 °C for 48 h. Milli Q water (50 mL) was added and the solution was extracted with DCM (3 x 40 mL). The organic phase was dried over $MgSO_4$ and the solvent evaporated under reduced pressure. The crude product was purified via column chromatography (silica, DCM/MeOH: 99/1 → 95/5). The product was recrystallized in EtOH, washed with EtOH and dried under vacuum to yield the target molecule.

Molecular formula: $C_{11}H_{11}N_3O_2$

Yield: 60 % (1.081 g, 4.98 mmol)

^{1}H-NMR (500 MHz, DMSO-D6) δ = 7.70 (d, J = 9.2 Hz, 2H), 6.90 (d, J = 9.2 Hz, 2H), 2.69 (s, 3H), 2.42 (s, 3H)

^{13}C-NMR (126 MHz, DMSO-D6) δ = 168.2, 160.6, 153.2, 145.6, 131.5, 124.2, 115.9, 11.8, 11.3

MS (*m/z*): (ESI, MeOH) Calculated for $[C_{11}H_{12}N_3O_2]^+$: 218.0924; found 218.09014

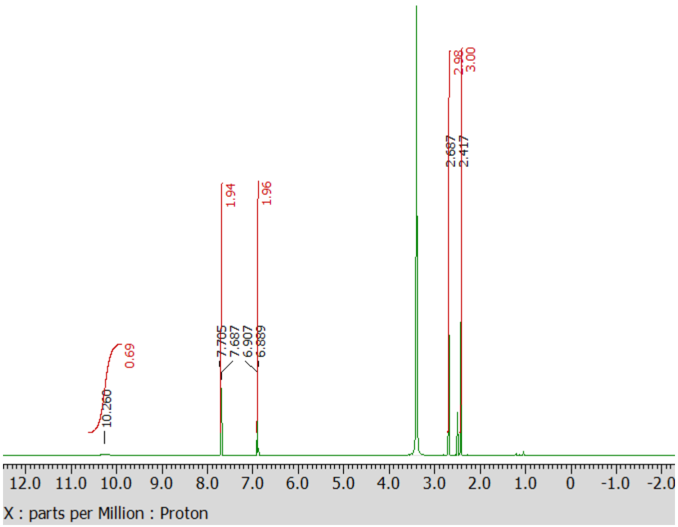

Supplementary Figure S2: ^{1}H-NMR spectrum of AIZ-OH.

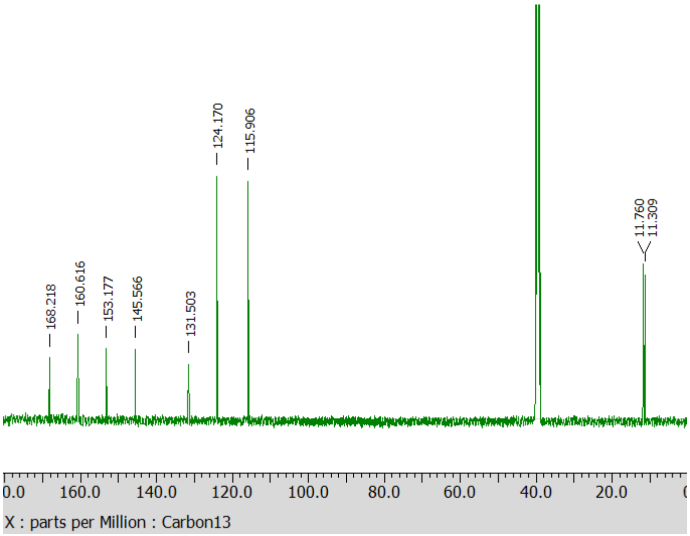

Supplementary Figure S3: ^{13}C-NMR spectrum of AIZ-OH.

Synthesis 2: 3,3'-Dihydroxyazobenzene (m-OH-m-OH)

Synthesis of 3-((tert-butyldimethylsilyl)oxy)aniline

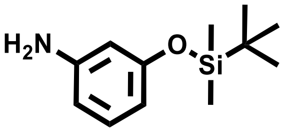

3-aminophenol (0.800 g, 7.33 mmol, 1.0 eq) and triethylamine (3 mL, 21.5 mmol, 2.9 eq) were suspended in DCM (18 mL). TBDMS-Cl (1.237 g, 8.21 mmol, 1.1 eq) was added and the mixture stirred at room temperature overnight. The reaction was quenched with water and the phases separated. The organic solvent was evaporated under reduced pressure. The crude product was purified by column chromatography (silica, DCM) to yield the desired compound.

Molecular formula: $C_{12}H_{21}NOSi$

Yield: 89 % (1.462 g, 6.54 mmol)

^{1}H-NMR (500 MHz, $CDCl_3$) δ = 6.99 (t, J = 8.0 Hz, 1H), 6.30 (dd, J = 8.0, 2.3 Hz, 1H), 6.26 (dd, J = 8.0, 2.3 Hz, 1H), 6.20 (t, J = 2.3 Hz, 1H), 0.98 (s, 9H), 0.19 (s, 6H)

^{13}C-NMR (126 MHz, $CDCl_3$) δ = 156.6, 147.6, 129.9, 110.4, 108.5, 107.1, 25.7, 18.2, -4.4

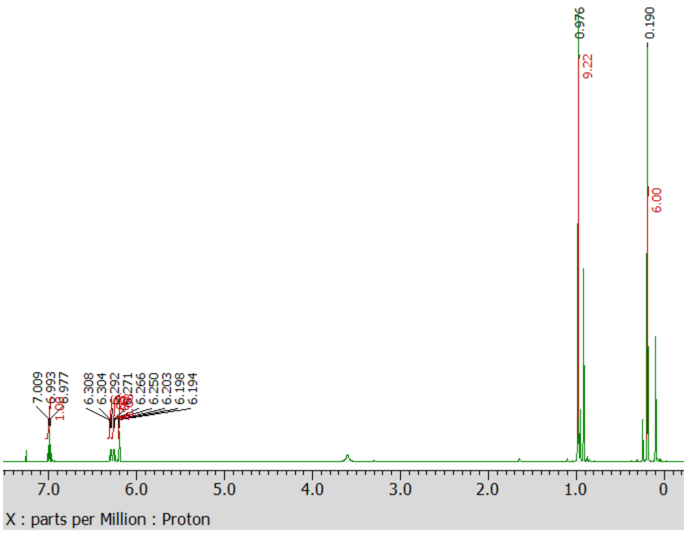

Supplementary Figure S4: ^{1}H-NMR spectrum of 3-((tert-butyldimethylsilyl)oxy)aniline.

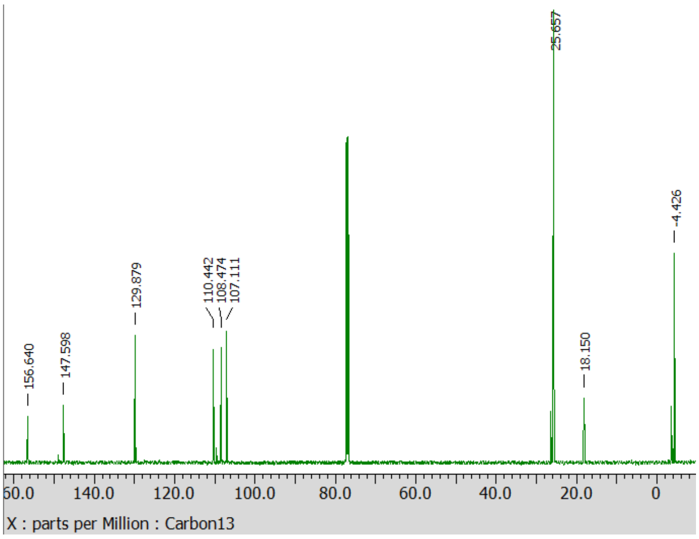

Supplementary Figure S5: ^{13}C-NMR spectrum of 3-((tert-butyldimethylsilyl)oxy)aniline.

Synthesis of 3,3'-di-((tertbutyl-dimethylsilyl)oxy) azobenzene

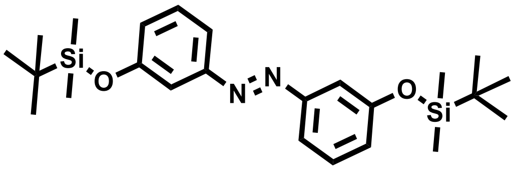

3-((tert-butyldimethylsilyl)oxy)aniline (1.321 g, 5.913 mmol, 1.0 eq), $KMnO_4$ (2.619 g, 16.57 mmol, 2.8 eq) and $FeSO_4$*7 H_2O (2.627 g, 9.45 mmol, 1.6 eq) were dispersed in DCM (25 mL) and refluxed overnight at 40 °C. The dark brown mixture was cooled to room temperature, filtered through silica to yield a red solution. After evaporation of the solvent under reduced pressure the crude product was purified via column chromatography (silica, DCM) to obtain the desired compound.

Molecular formula: $C_{24}H_{38}N_2O_2Si_2$

Yield: 18 % (0.235 g, 0.531 mmol)

^{1}H-NMR (500 MHz, $CDCl_3$) δ = 7.53 (d, J = 8.0 Hz, 2H), 7.38-7.35 (m, 4H), 6.96 (d, J = 8.0 Hz, 2H), 1.01 (s, 18H), 0.25 (s, 12H)

^{13}C-NMR (126 MHz, $CDCl_3$) δ = 156.4, 153.9, 129.6, 122.8, 116.7, 113.7, 25.7, 18.2, -4.4

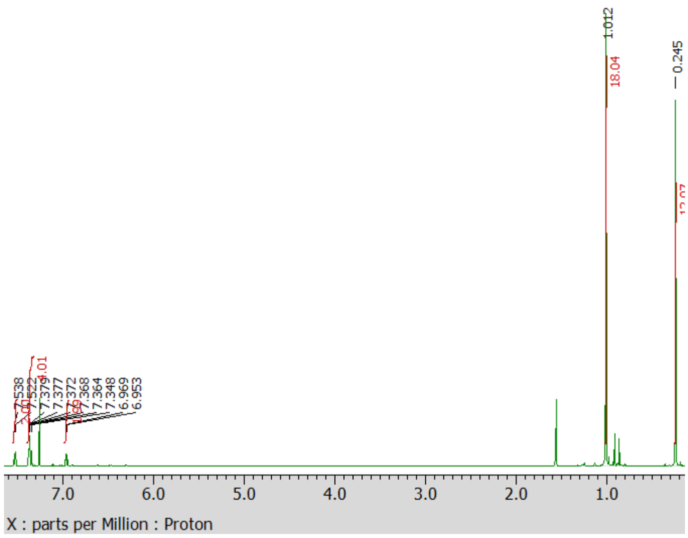

Supplementary Figure S6: ^{1}H-NMR spectrum of 3,3'-di-((tertbutyl-dimethylsilyl)oxy) azobenzene.

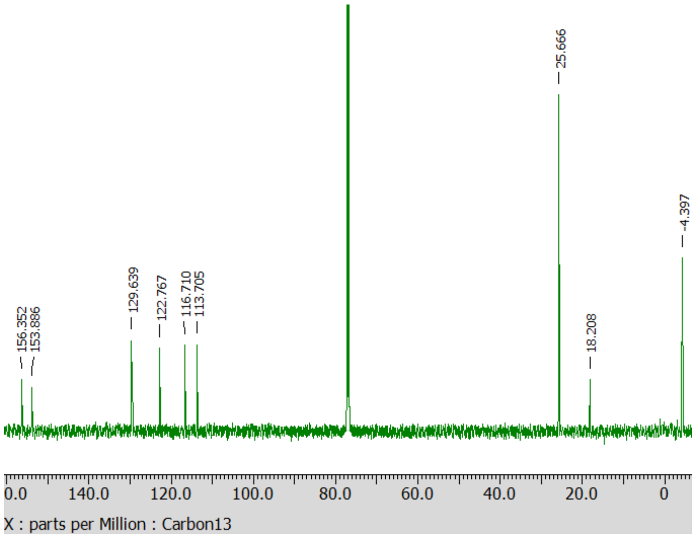

Supplementary Figure S7: ^{13}C-NMR spectrum of 3,3'-di-((tertbutyl-dimethylsilyl)oxy) azobenzene.

Synthesis of 3,3’-Dihydroxyazobenzene

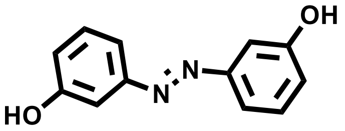

The previously synthesized 3,3’-di-((tertbutyl-dimethylsilyl)oxy) azobenzene (0.229 g, 0.517 mmol, 1.0 eq) was dissolved in THF (5 mL) and TBAF (1.1 mL, 1 M, 1.1 mmol, 2.1 eq) was added. The mixture was stirred at room temperature for 1h. The reaction was quenched with NH4Cl. After addition of 15 mL H_2O, the product was extracted with EtOAc (3x 20 mL) and the solvent of the combined organic phases was evaporated under reduced pressure. Column chromatography (silica, DCM/MeOH: 98/2) and recrystallisation in EtOH/H_2O yielded the target molecule.

Molecular formula: $C_{12}H_{10}N_2O_2$

Yield: 45 % (0.05 g, 0.233 mmol)

^{1}H-NMR (500 MHz, MeOD-D4) δ = 7.40-7.37 (m, 2H), 7.34 (t, J = 8.0 Hz, 2H), 7.28 (t, J = 2.0 Hz, 2H), 6.93 (dq, J = 7.7, 1.2 Hz, 2H)

^{13}C-NMR (126 MHz, MeOD-D4) δ = 159.5, 155.3, 130.9, 119.3, 116.6, 108.8

MS (*m/z*): (ESI, MeOH) Calculated for $[C_{12}H_{11}N_2O_2]^+$: 215.0815; found 215.07936

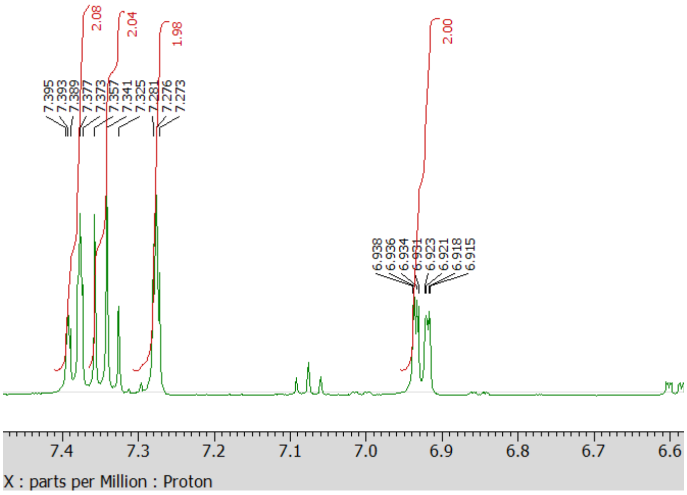

Supplementary Figure S8: ^{1}H-NMR of 3,3’-Dihydroxyazobenzene.

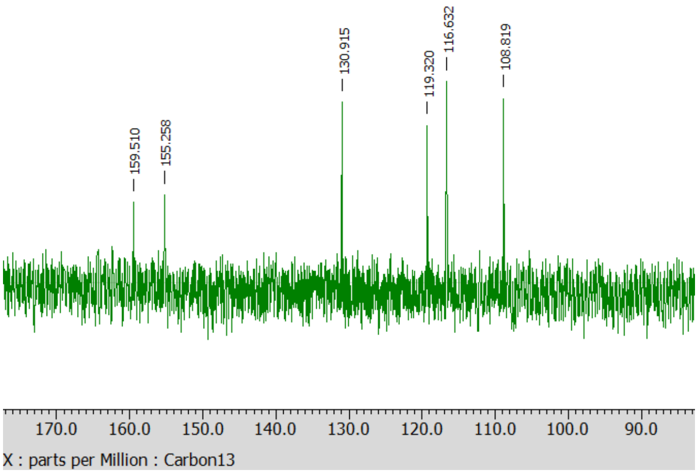

Supplementary Figure S9: ^{13}C-NMR spectrum of 3,3’-Dihydroxyazobenzene.

Synthesis 3: 3'-Carboxy-4-dimethylaminoazobenzene (DMA-m-COOH)

DMA-m-COOH was synthesized by following a method reported by Tushar S. Basu Baul, Dhrubajyoti Dutta et al.[2]

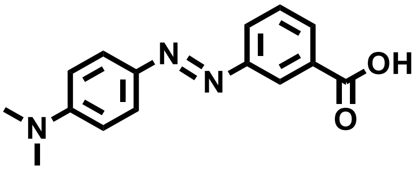

m-aminobenzoic acid (1 g, 7.29 mmol) in a mixture of concentrated HCl (0.7 mL) and water (2 mL) was diazotized with cold $NaNO_2$ solution (0.550 g, 8 mmol, 1.1 mL water). A cold solution of N,N-dimethyl aniline (1.3 g, 10.7 mmol) was then added fairly rapidly to the cold diazonium salt solution and held at 0–5 °C in an ice-bath, with vigorous stirring. A deep-red precipitate developed immediately, and the stirring was continued for 15 min and then an aqueous solution of sodium acetate (1.1 g in 1.5 mL water) was added and stirring was continued for 1 h. The reaction mixture was kept overnight in a refrigerator, followed by 2 hours at room temperature. Sodium hydroxide solution (1.5 mL, 20%) was added to have a distinct odor of dimethylaniline and the reaction mixture was kept at room temperature for 1 h. The precipitate was filtered, washed with water, then acetic acid (3 mL, 10%) to remove excess dimethylaniline, again washed several times with water, and finally with methanol and then dried in air. The crude product was washed thoroughly with hexane to remove any tar-like material and then dissolved in toluene. Several recrystallizations from methanol yielded dark-orange crystalline product.

Molecular formula: $C_{15}H_{15}N_3O_2$

Yield: 60%

^{1}H-NMR (500 MHz, $CDCl_3$): δ 8.56 (s, 1H), 8.09 (m, 2H), 7.91 (dd, 2H), 7.58 (t, 1H), 6.77 (dd, 2H), 3.11 (s, 6H) ppm.

^{13}C NMR (125 MHz, $CDCl_3$): δ 153.45, 152.86, 143.60, 130.71, 129.28, 127.23, 127.12, 125.46, 124.28, 111.61, 40.46 ppm.

ESI-MS (*m/z*): [M+H]+ Calculated 270.1237 ; found 270.1234

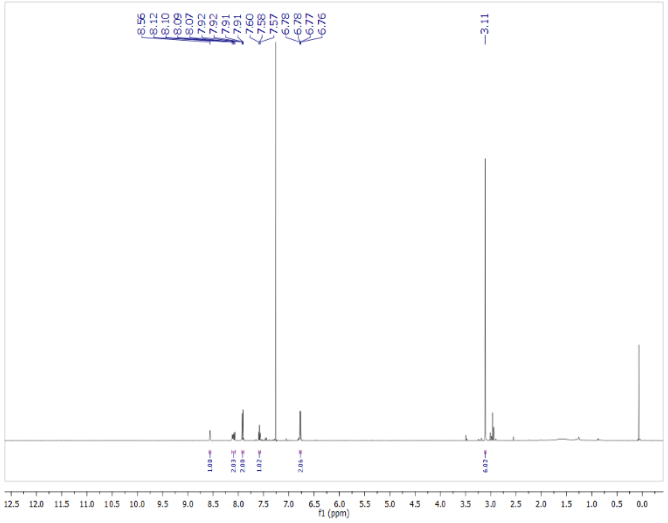

Supplementary Figure S10: ^{1}H-NMR spectrum of DMA-m-COOH

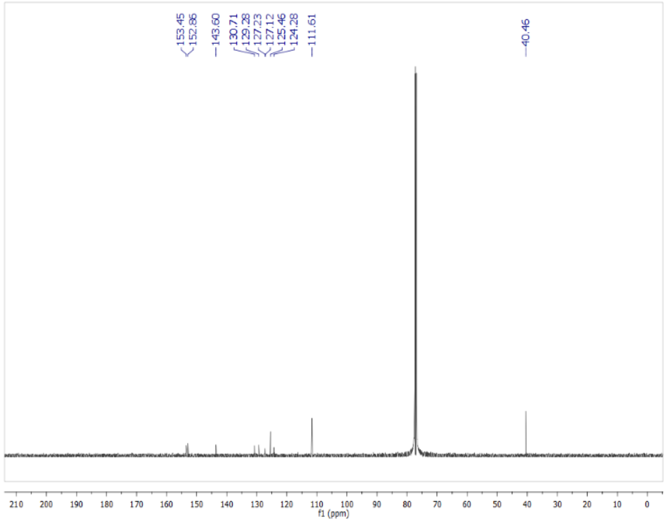

Supplementary Figure S11: ^{13}C NMR spectrum of DMA-m-COOH

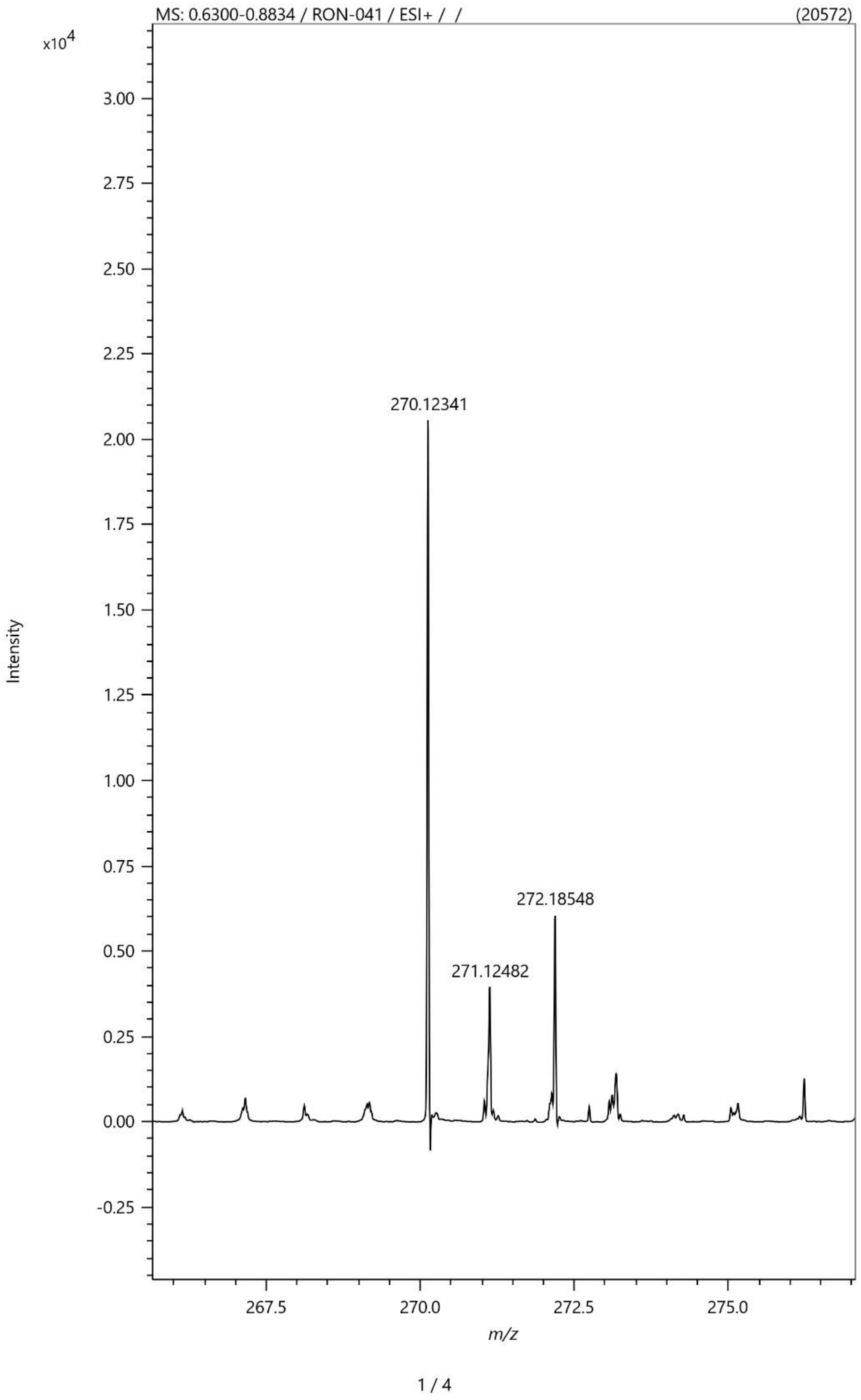

Supplementary Figure S12: MS data of DMA-m-COOH

Synthesis 4: 4-(Pyridin-4-yldiazenyl)phenol (Pyr-OH)

DMA-m-COOH was synthesized by following a method reported by Huidan Lu et al.[3]

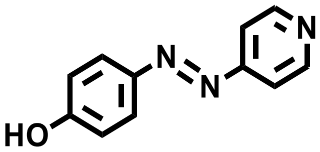

4-Hydroxyazopyridine was prepared from 4-aminopyridine and phenol. To a solution of $NaNO_2$ (4.0 g) in water (20 mL), phenol (5 g) in 10 % NaOH solution (45 mL) was added. At 0 °C, the mixture was added to a solution of 4-aminopyridine (6 g) in hydrochloric acid (25 mL of concentrated HCl:16 mL of water) under stirring in 10 min. The solution was then adjusted to pH about 6 using saturated aqueous Na_2CO_3 solution to give yellow precipitates. The product was then filtrated out of solution and dried in air.

Molecular formula: $C_{11}H_9N_3O$

Yield: ~75 %

^{1}H-NMR (500 MHz, METHANOL-D4) δ = 8.69 (d, J = 6.3 Hz, 2H), 7.90 (d, J = 9.2 Hz, 2H), 7.76 (d, J = 6.3 Hz, 2H), 6.94 (d, J = 9.2 Hz, 2H)

MS (*m/z*): (ESI, MeOH) Calculated for $[C_{11}H_{10}N_3O]^+$: 200.0818; found 200.0768

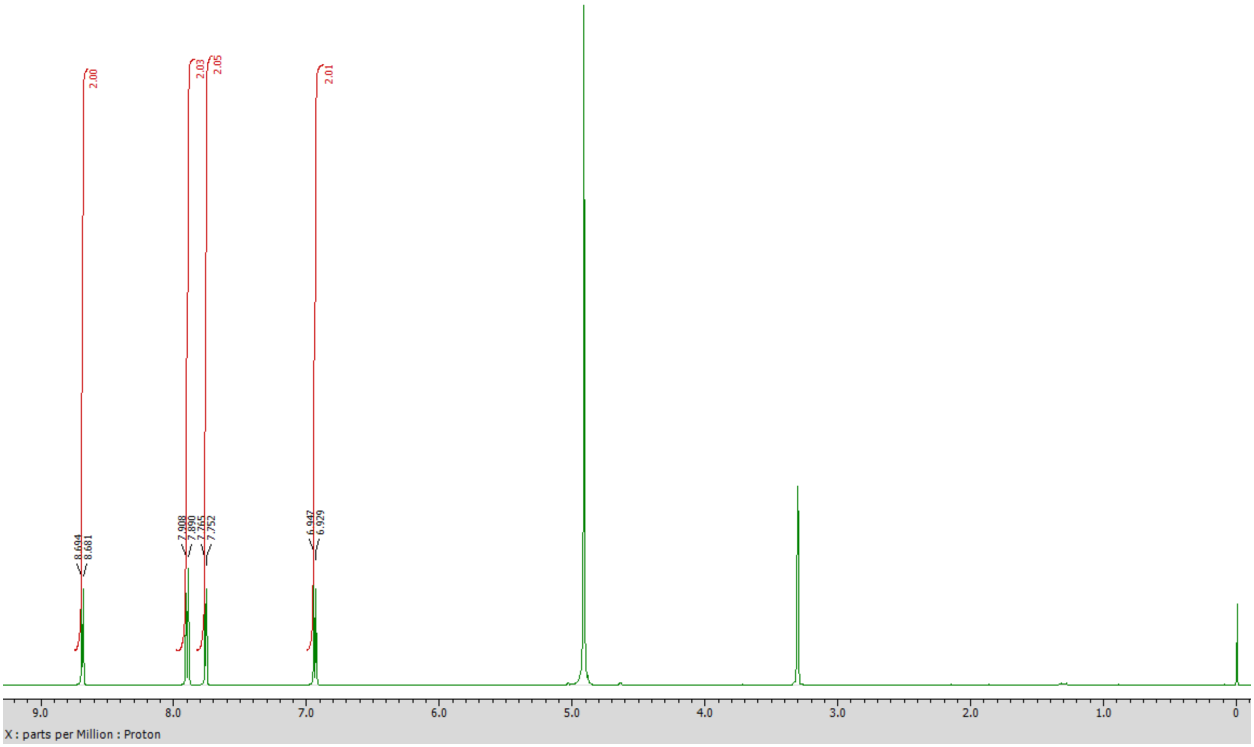

Supplementary Figure S13: ^{1}H-NMR spectrum of Pyr-OH.

Synthesis 5: 4-(4-Ethylphenylazo)phenol (2PAP)

2PAP synthesis was carried out following the procedure reported by S. Suwanprasop et al.[4]

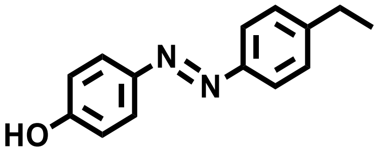

Molecular formula: $C_{14}H_{14}N_2O$

^{1}H-NMR (500 MHz, CHLOROFORM-D) δ = 7.83 (dd, J = 14.7, 8.7 Hz, 4H), 7.33 (d, J = 8.0 Hz, 2H), 6.90 (d, J = 9.2 Hz, 2H), 5.94 (s, 1H), 2.72 (q, J = 7.6 Hz, 2H), 1.28 (t, J = 7.7 Hz, 3H)

MS (*m/z*): (ESI, MeOH) Calculated for $[C_{14}H_{15}N_2O]^+$: 227.1179; found 227.1135

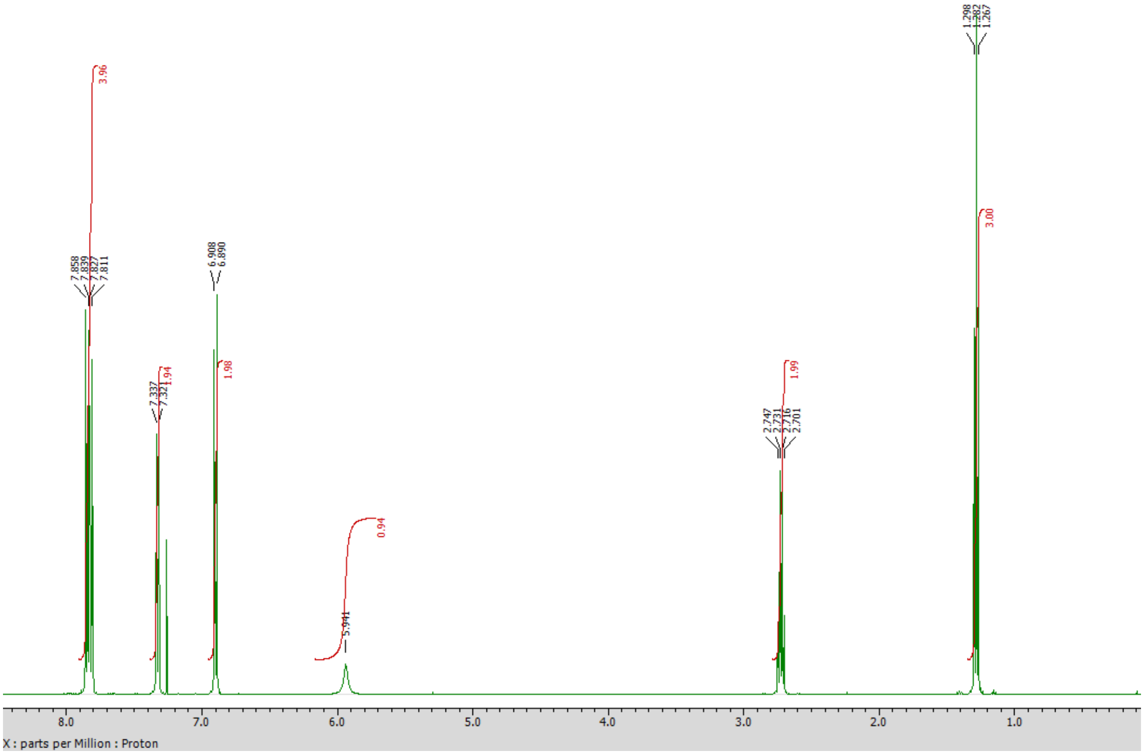

Supplementary Figure S14: ^{1}H-NMR spectrum of 2PAP

Thin film sample preparation and isomerisation wavelengths

Polymer thin film samples containing azobenzene derivatives were prepare by dissolving the polymer and azobenzene in either ethanol (EtOH), ethyl acetate (EtOAC), dimethyl formamide (DMF), a mixture of ethanol and DMF or a mixture of ethanol and ethyl acetate. The choice of solvent was primarily due to the solubility of the polymer. In cases where the azobenzene did not dissolve in ethanol DMF or EtOAc was added to help dissolve the azobenzene. For some azobenzenes pure DMF had to be used to dissolve them enough for film preparation. Supplementary Table S1 lists all the prepared stock solutions with details. Used polymers were poly-4-vinylpyridine (P4VP), poly(methyl methacrylate) (PMMA), polystyrene (PS) and ethyl cellulose (EC). Note that for EC the molar ratio was calculated for number of azobenzene molecules per hydroxy groups in the polymer, rather than per monomeric unit. Structures for the azobenzenes can be found in the article, except for 3-3'-dihydroxyazobenzen (m-OH-m-OH).

After preparing the solutions thin film were fabricated by spin coating at 1000 rpm for 30-40 seconds. 100-200 µl of stock solution was deposited on a glass substrate before starting the spinning. After spinning the excess solvent was evaporated from the film by heating the films on a 60 °C hotplate for 5-10 minutes. For films made from pure DMF stock solution excess solvent was evaporated by keeping the films under vacuum at 40 °C overnight.

Isomerisation wavelength for humidity sensitivity experiments were chosen based on the absorption maximum of the azobenzene. Excitation wavelength was chosen to be close to the absorption maximum but preferably slightly longer wavelength. When possible, the LED used for excitation was chosen so that there was minimal interference with the monitoring wavelength window, for example using 385 nm LED for excitation when monitoring was done at 360-370 nm.

Supplementary Table S1: Stock solution prepared for the film fabrication. Used polymer, azobenzene and solvent, molar ratios (mol:mol) describing number of azobenzene molecules per monomeric units in polymer, azobenzene mass-% in the dry film calculated from actual weighed masses, and concentration of polymer + azobenzene in solvent (total dry concentration).

Azobenzene/polymer	Solvent	Molar ratio	Azobenzene m-%	Total dry concentration [mg/ml]	Photoexcitation wavelength [nm]
2PAP/P4VP	EtOH	1:8	22.2	25	385
CN-OH/P4VP	EtOH	1:8	20.8 (21.5)[e]	25 (23.9)[e]	385
CN-OH/P4VP	EtOH	1:4	35.1	29.6	385
CN-OH/P4VP	EtOH	1:1	67.2	38,6	385
DMA-OH/P4VP	EtOH/DMF[a]	1:8	22.3	23.8	435
OH-OH/P4VP	EtOH	1:8	20.4	25	385
AIZ-OH/P4VP	EtOH	1:8	19.1	25	365
Pyr-OH/P4VP	EtOH/DMF[b]	1:8	19.1	22.7	385
DMA-COOH/P4VP	DMF	1:8	24.2	60	460
DMA-m-COOH/P4VP	EtOH	1:8	24.4	25	460
DMA-NH_2/P4VP	EtOH/EtOAc[c]	1:8	22.0	25	435
NO_2-NH_2/P4VP	EtOH/EtOAc[c]	1:8	22.3	25	460
NH_2-NH_2/P4VP	EtOH/DMF[a]	1:8	20.0	23.8	435
CN-OH/PMMA	EtOAc	1:8	21.3	25	385
CN-OH/EC	EtOH	1:8[d]	10.8	25	385
m-OH-m-OH/P4VP	EtOH	1:8	20.0	25	365

[a] 50 µl of DMF per 1 ml of EtOH

[b] 100 µl of DMF per 1 ml of EtOH

[c] 1:1 mixture

[d] Molar ratio for EC is in term of azobenzene molecules per hydroxy groups in the polymer, not azobenzene molecules per monomeric unit.

[e] for absorption and thermal isomerisation kinetic measurement values outside of bracket, for DVS and QCM measurements values inside brackets

Polarized optical microscopy images of films

Film stability of CN-OH/P4VP films with different CN-OH content were compared to illustrate how high azobenzene content can easily lead to phase separation. The comparison was done using a polarized optical microscope (POM), with out and with the cross polarizer in place. Without cross polarizer one can see aggregated areas as dark spots and with the cross polarizer these areas have birefringence, indicating crystallised azobenzene. The film with 1:8 CN-OH content was imaged a year after fabrication and there was only a small number of aggregates seen throughout the film. The film with 1:1 CN-OH content was imaged only 4 months after fabrication and it shows significantly more aggregates throughout the film. These aggregates are also smaller than those seen in the 1:8 film. The fact that 1:1 CN-OH content lead to such significant difference in the stability of the film is an important point of consideration when developing new sensor materials.

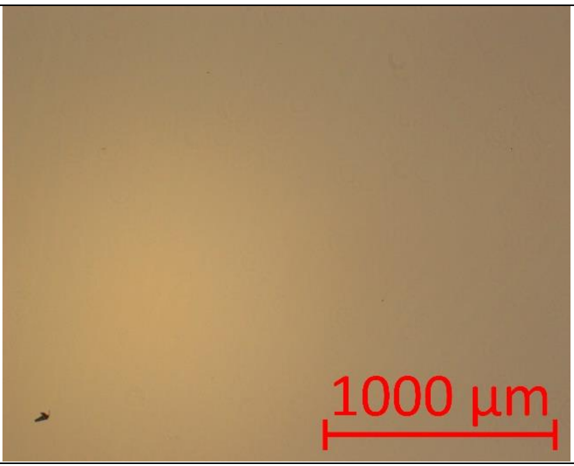

1:8 CN-OH:P4VP
12 months old film

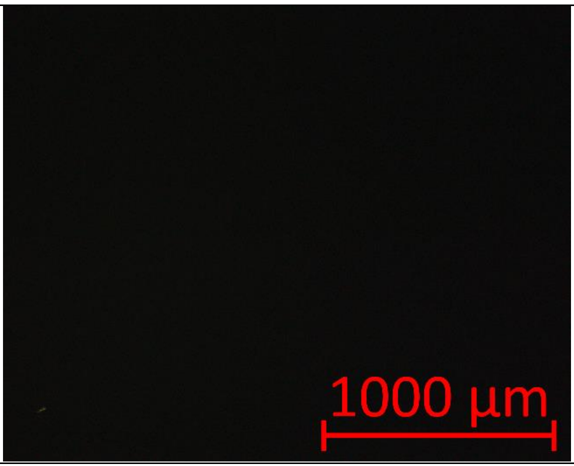

1:8 CN-OH:P4VP
12 months old film
With cross polarisers

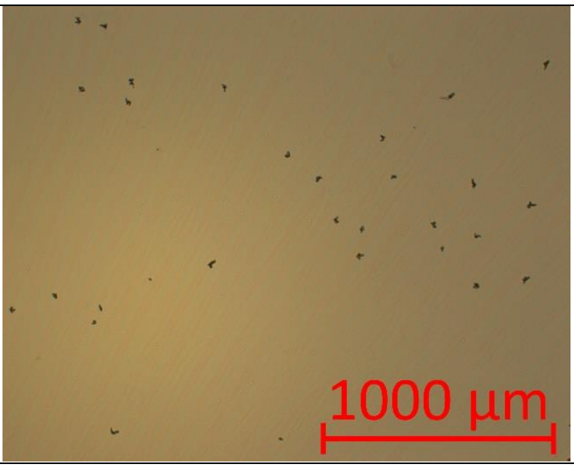

1:1 CN-OH:P4VP
4 months old film

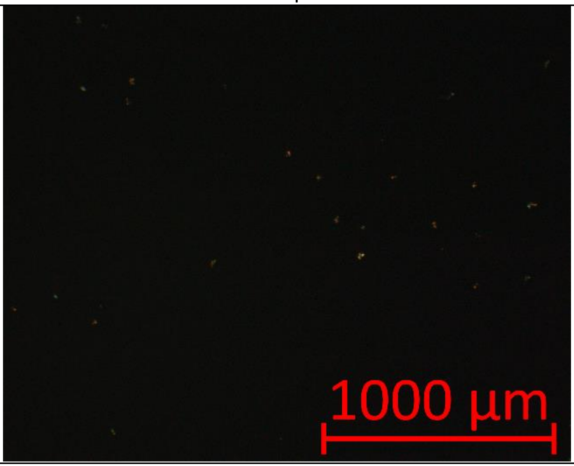

1:1 CN-OH:P4VP
4 months old film
With cross polarisers

Supplementary Figure S15: POM images of CN-OH/P4VP films with 1:8 (top) and 1:1 (bottom) CN-OH content. Imaged in bright field mode (left column) and with cross polarizers in place (right column).

Lifetime of studied azo-compounds in solution

Experimental:

To determine the cis-lifetimes (τ) of various azo-photoswitches in solution, 25 µM solutions of the respective photoswitch in dry THF were prepared. To minimize water absorption by exposure to humidity in ambient air, during each step of preparation (and when filling into the cuvette) an argon or nitrogen atmosphere was established by purging the gas phase of each container. For one compound (CN-Azo-OH) humidity dependent relaxation studies were conducted, by adding water to create concentrations between 10 and 1000 ppm water inside the solution. The measurement solutions were kept at a constant temperature of 25 °C and irradiated with UV (or blue) light until the PSS was reached. Immediately after the irradiation was stopped, the measurement series was started to record a spectrum after different times, with increasing interval times in between measurements. The absorbance at the initial absorbance maximum was plotted against time and an exponential (Arrhenius) fit (see below) was used to determine relaxation constant R_0.

$$y = y_0 + A \cdot exp(-R_0 \cdot t)$$

The lifetime was then determined by the following relation:

$$\tau = 1 / R_0$$

Discussion:

The cis-lifetimes of the photoswitches in focus at 25 °C were also studied in solution state, with THF as solvent. By plotting the time dependent absorbance of each photoswitch after irradiation and performing an exponential fit, the relaxation constants and thus the lifetimes were determined. Supplementary Table S2 shows the obtained results. The lifetimes measured in THF are comparable to lifetimes measured or predicted in P4VP films at relative humidities below 35 % for all compounds. For some the lifetime in dry THF is close to predicted lifetime in P4VP at close to 0 % RH and for some at close to 30 % RH. It is interesting that for some azobenzenes the predicted lifetime in completely dry film is close to lifetime in dry solvent, since one would expect the isomerisation to be restricted in solid phase compared to solution. The restricted movement does however not show in the lifetime but rather the stretched exponential relaxation in films compared to first-order exponential relaxation in solution.

Besides this, the behaviour of CN-Azo-OH was more thoroughly investigated, repeating the measurement two times (always using a freshly prepared solution). As the obtained τ range between 4237 and 6579 s, it is assumed that these relaxation of this azobenzene is highly sensitive to the environmental conditions and thus trace amounts of water inside the solution also being capable of drastically changing the behaviour of the molecule in study. To further analyse this intriguing finding, we performed an additional series of measurements, this time adding trace amounts of water (10 to 1000 ppm) into the solution and performing the same measurements again. In order to minimize environmental deviations, the solutions were prepared from the same stock solution and conducted on the same day. Upon increasing the water content, a drastic lifetime decrease was observed, from nearly 36 min at 10 ppm water content to about 4 minutes with 1000 ppm. The most pronounced change occurs between 10 and 100 ppm water, as there the lifetime decreases already to 575 s (~10 min), showing that at already fairly low water concentrations, a significant impact takes place.

Supplementary Table S2: Results of cis-lifetime measurements of different photoswitches in THF and Toluene at 25 °C

Photoswitch	Solvent	Concentration	Added water-content	Relaxation constant	R^2	*cis*-lifetime (τ)
CN-Azo-OH	THF	25 µM		0.000236	0.99986	4237 s
CN-Azo-OH	THF	25 µM		0.000152	0.99998	6579 s
CN-Azo-OH	THF	25 µM	10 ppm	0.000465	0.99811	2151 s
CN-Azo-OH	THF	25 µM	100 ppm	0.00174	0.99288	575 s
CN-Azo-OH	THF	25 µM	1000 ppm	0.0039	0.99523	256 s
DMA-Azo-OH	THF	25 µM		0.000345	0.99998	2899 s
4,4'-HO-Azo-OH	THF	25 µM		0.0001389	0.99995	7199 s
H2N-Azo-NH2	THF	25 µM		0.00123	0.99992	813 s
H2N-Azo-NO2	THF	25 µM		0.67022	0.98804	1.5 s
Pyr-Azo-OH	THF	25 µM		0.0208	0.99074	48 s
AIZ-OH	THF	25 µM		0.0000114	0.99985	87719 s
DMA-Azo-p-COOH	THF	25 µM		0.000583	0.99985	1715 s
DMA-Azo-m-COOH	THF	25 µM		0.000757	0.9994	1321 s

Spectra and data plots:

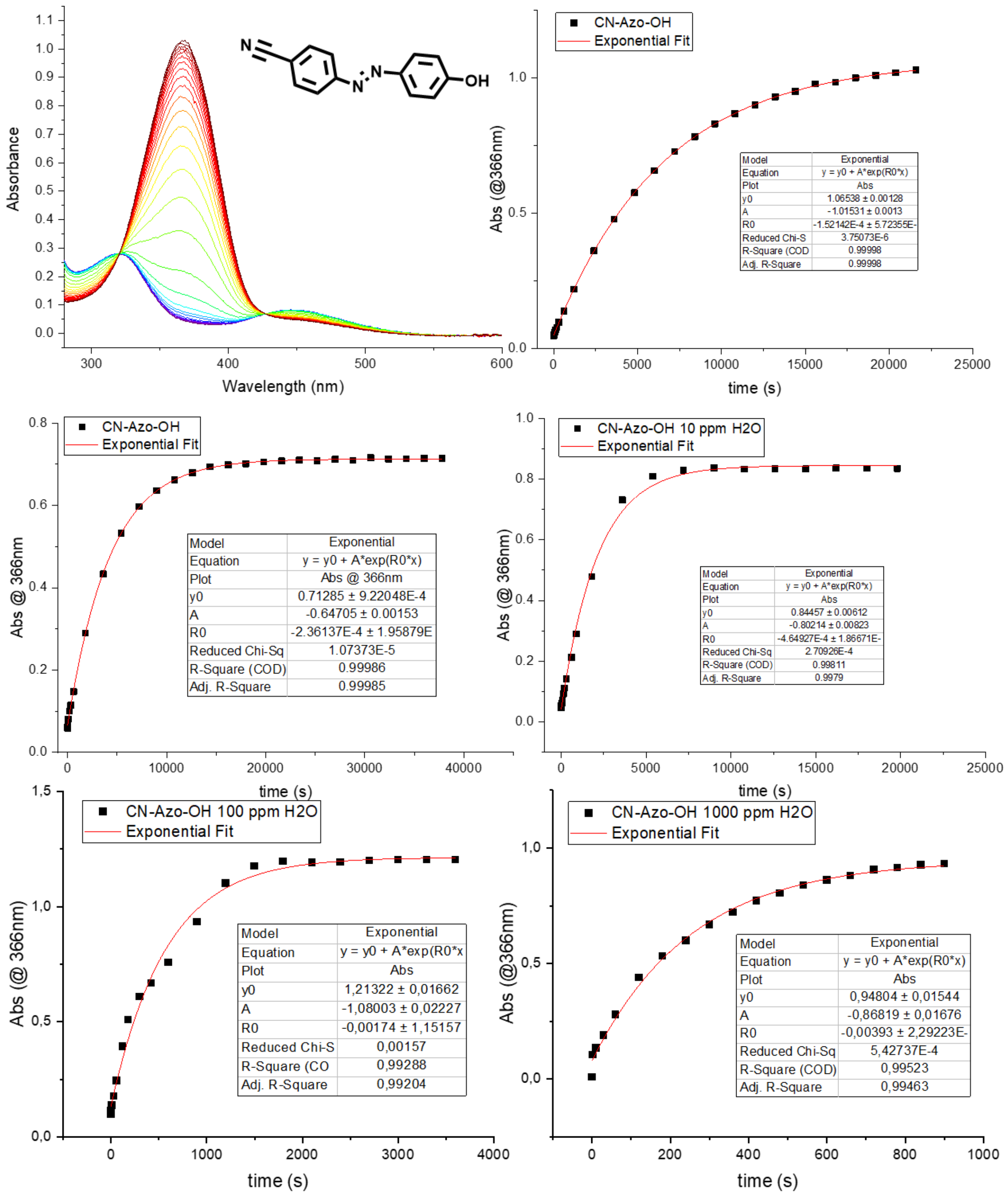

Supplementary Figure S16: Absorbance spectrum and time-dependent absorbance of AIZ-OH at 340 nm at 25 °C in THF.

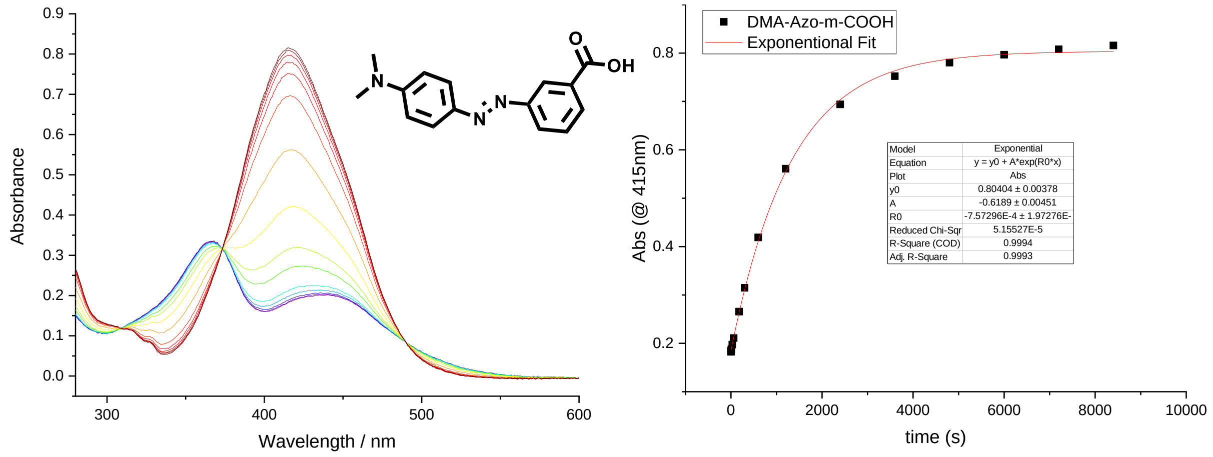

Supplementary Figure S17: Absorbance spectrum and time-dependent absorbance of DMA-Azo-m-COOH at 415 nm at 25 °C in THF.

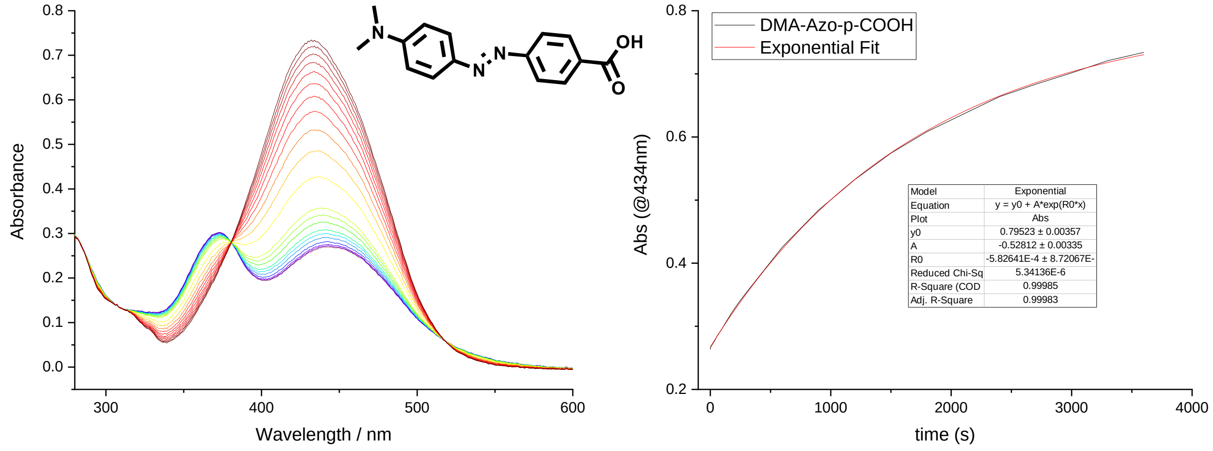

Supplementary Figure S18: Absorbance spectrum and time-dependent absorbance of DMA-Azo-p-COOH at 434 nm at 25 °C in THF.

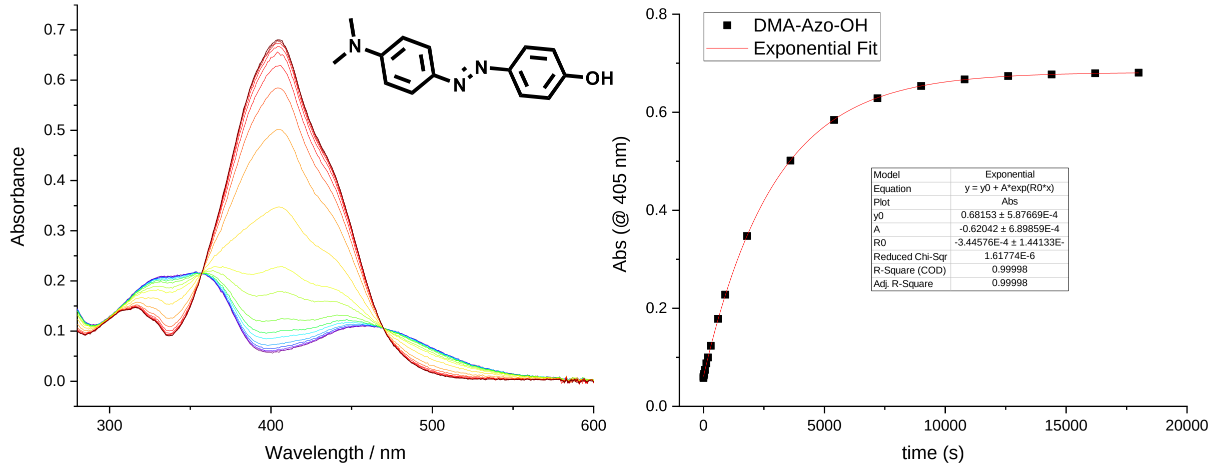

Supplementary Figure S19: Absorbance spectrum and time-dependent absorbance of DMA-Azo-OH at 405 nm at 25 °C in THF.

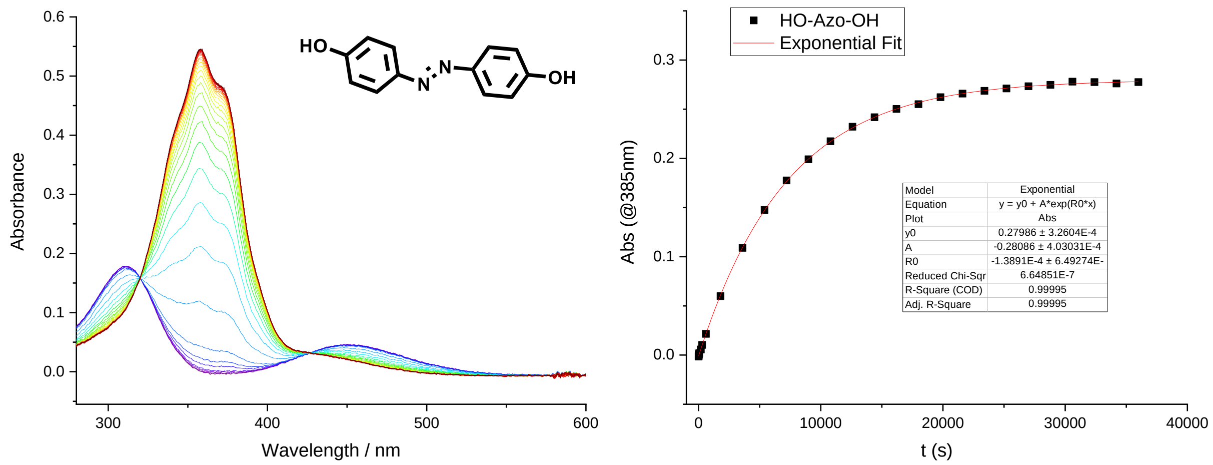

Supplementary Figure S20: Absorbance spectrum and time-dependent absorbance of HO-Azo-OH at 385 nm at 25 °C in THF.

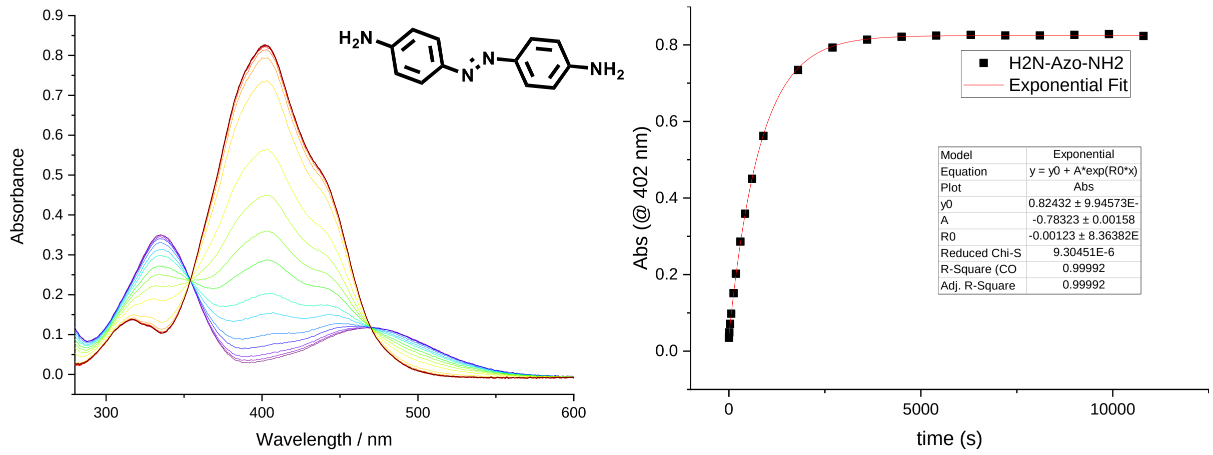

Supplementary Figure S21: Absorbance spectrum and time-dependent absorbance of H_2N-Azo-NH_2 at 402 nm at 25 °C in THF.

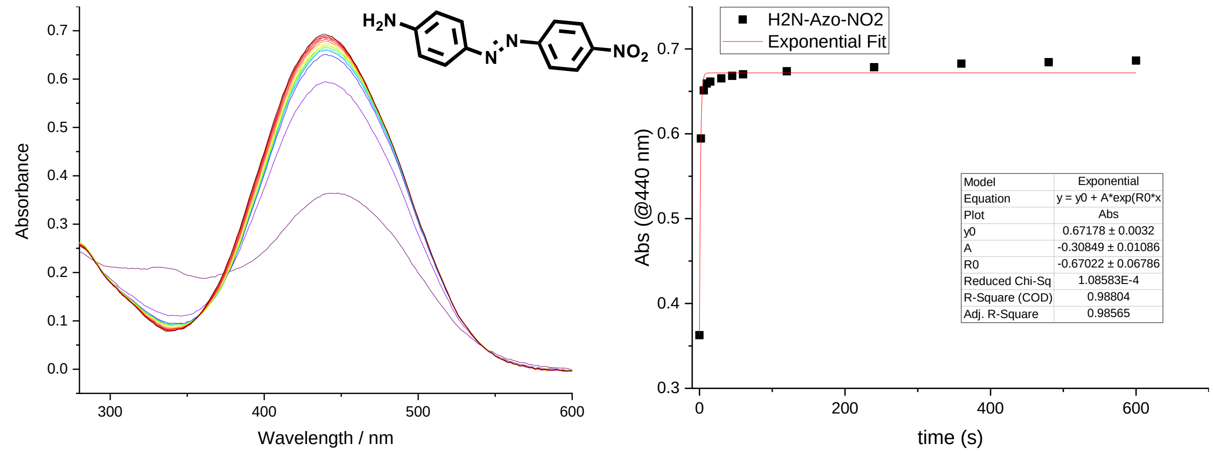

Supplementary Figure S22: Absorbance spectrum and time-dependent absorbance of H_2N-Azo-NO_2 at 440 nm at 25 °C in THF.

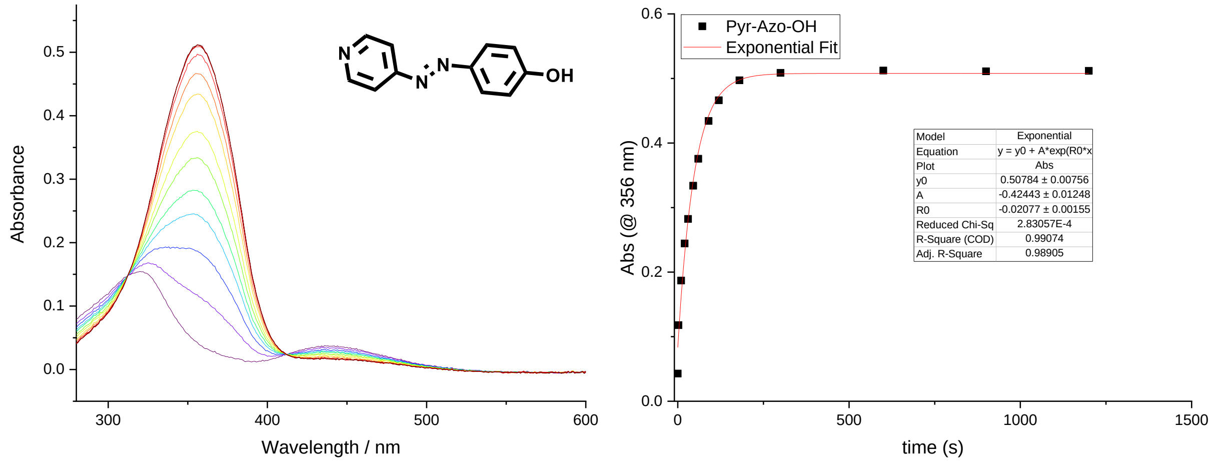

Supplementary Figure S23: Absorbance spectrum and time-dependent absorbance of Pyr-Azo-OH at 356 nm at 25 °C in THF.

Computational studies

The molecular geometry optimizations and energy calculations were performed at the wB97X/def2-TZVP level of theory using ORCA 5.0.4. Frequency calculations were performed for all optimized geometries at the same level of theory to ensure that the obtained geometries were true minimum energy geometries (i.e. no imaginary frequencies). The conductor-like polarizable continuum method (CPCM) was used to account for solvent effects. The calculated energies compared to the *cis*-azo tautomer are listed in Supplementary Table S3.

Supplementary Table S3: DFT calculations of state energies in THF

ωB97X/def2-TZVP	Energies relative to *cis*-isomer in THF (kcal/mol)		
**Sample**	*cis*-hydrazone	hydrazone transition state	inversion transition state
2PAP	2.5	4.0	29.1
CN-OH	1.8	2.1	22.7
DMA-OH	1.4	7.0	30.7
OH-OH	1.6	5.9	31.5
Aiz-OH	1.3	7.4	31.5
Pyr-OH	1.0	2.2	22.1
NO_2-NH_2	9.9	1.3	20.4
NH_2-NH_2	13.3	2.9	32.0
DMA-NH_2	13.2	3.1	31.8

AFM imaging of thin films

To have an accurate idea of the thickness of the P4VP thin films AFM images were taken from a few select samples. A film without azobenzene was imaged as well as films fabricated from distinctly different solvents or mixtures. film with CN-OH was fabricated from ethanol solution, film with NO2-NH2 from 1:1 ethanol/ethyl acetate mixture and film with DMA-COOH from DMF solution. It is clear that addition of azobenzene significantly increases the film thickness, with pure P4VP film being only a third of the thickness of CN-OH/P4VP film. It is good to note that in the films with azobenzene the concentration of stock solution is expressed as the total of P4VP and azobenzene, meaning that in the film with azobenzene the concentration of P4VP is even smaller. Also, from the results it is clear that films fabricated from DMF is significantly thinner than from the other solutions. This makes sense given the weaker adhesion of DMF to the glass substrate. Because of this a higher concentration of azobenzene and P4VP in the DMF solution was required to get sufficient absorbance levels.

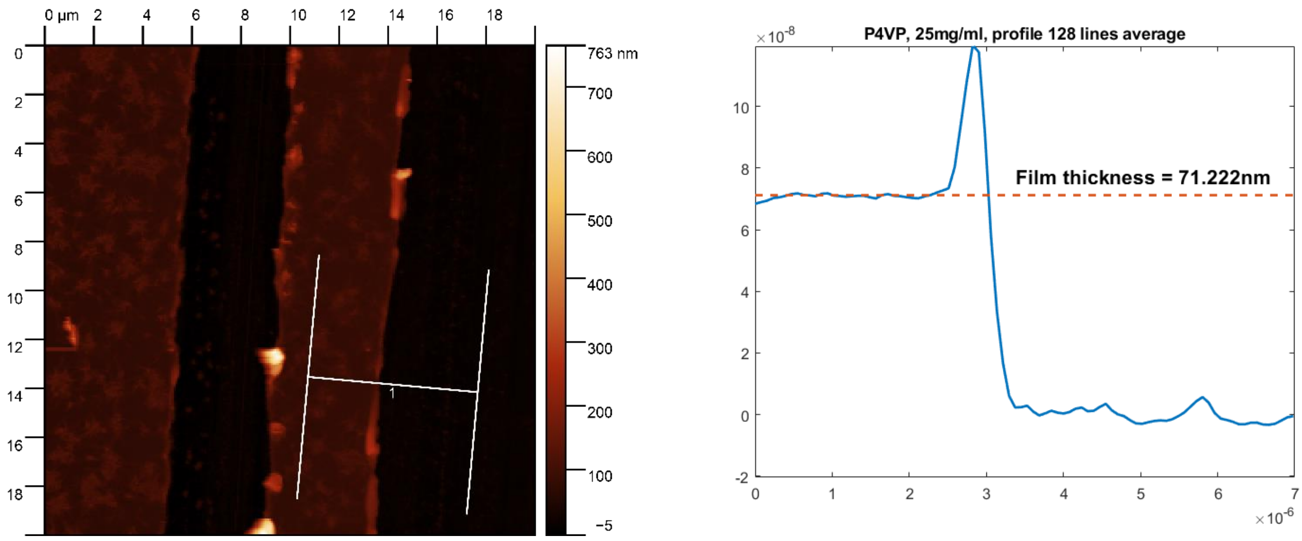

Supplementary Figure S24: AFM image of P4VP thin film spin coated from 25 mg/ml ethanol solution at 1000 rpm and the average side profile from 128 lines.

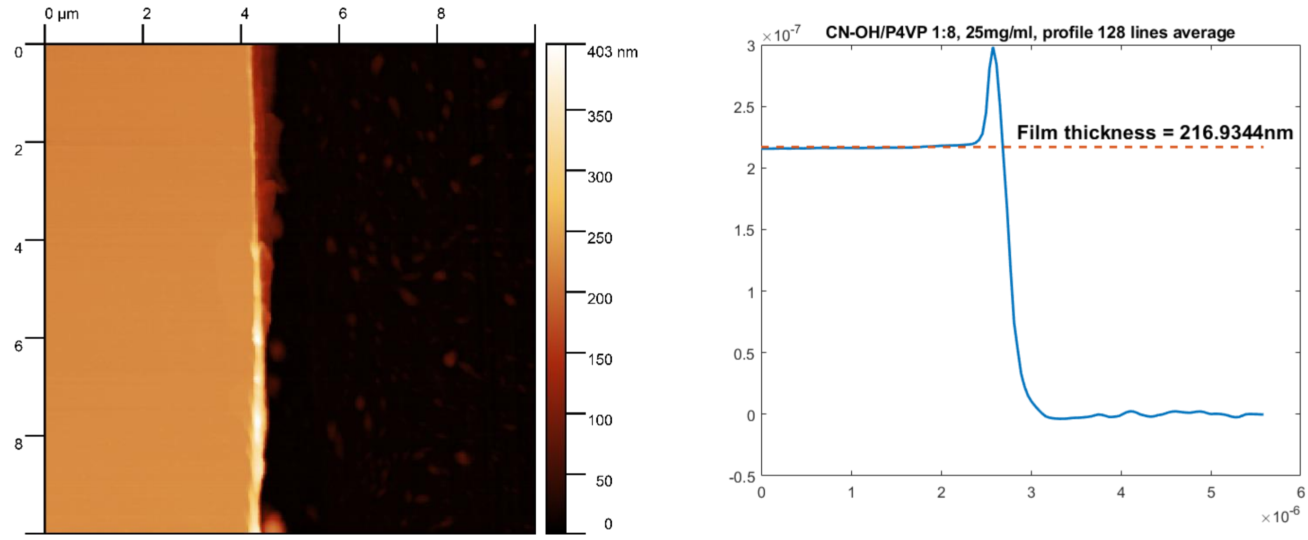

Supplementary Figure S25: AFM image of 1:8 CN-OH/P4VP thin film spin coated from 25 mg/ml ethanol solution at 1000 rpm and the average side profile from 128 lines.

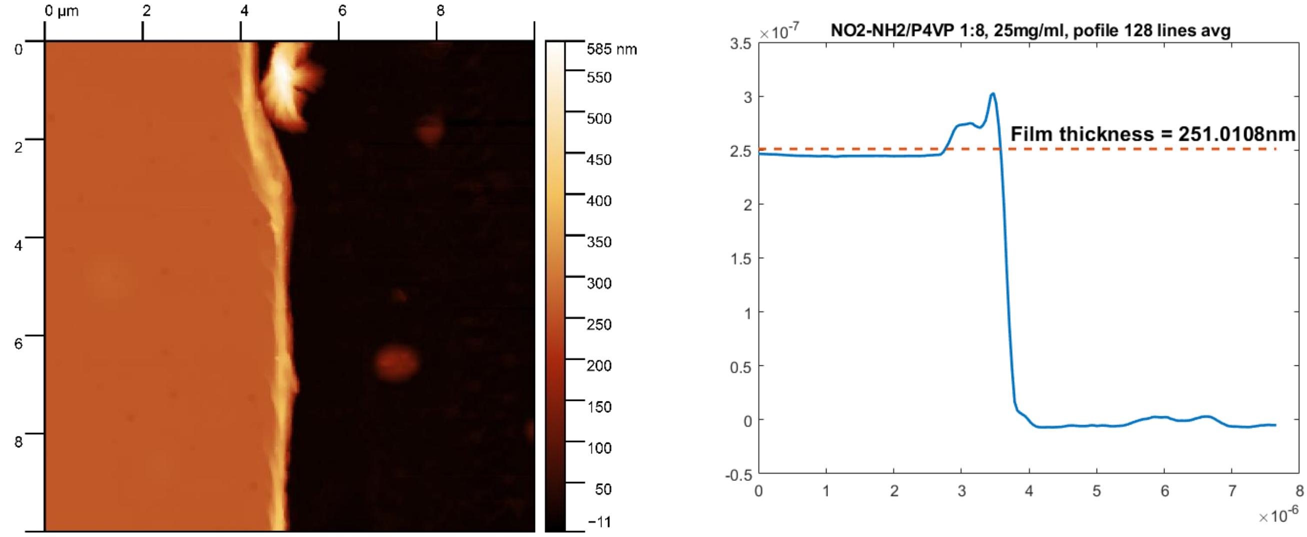

Supplementary Figure S26: AFM image of 1:8 NO_2-NH_2/P4VP thin film spin coated from 25 mg/ml 1:1 EtOH/EtOAc solution at 1000 rpm and the average side profile from 128 lines.

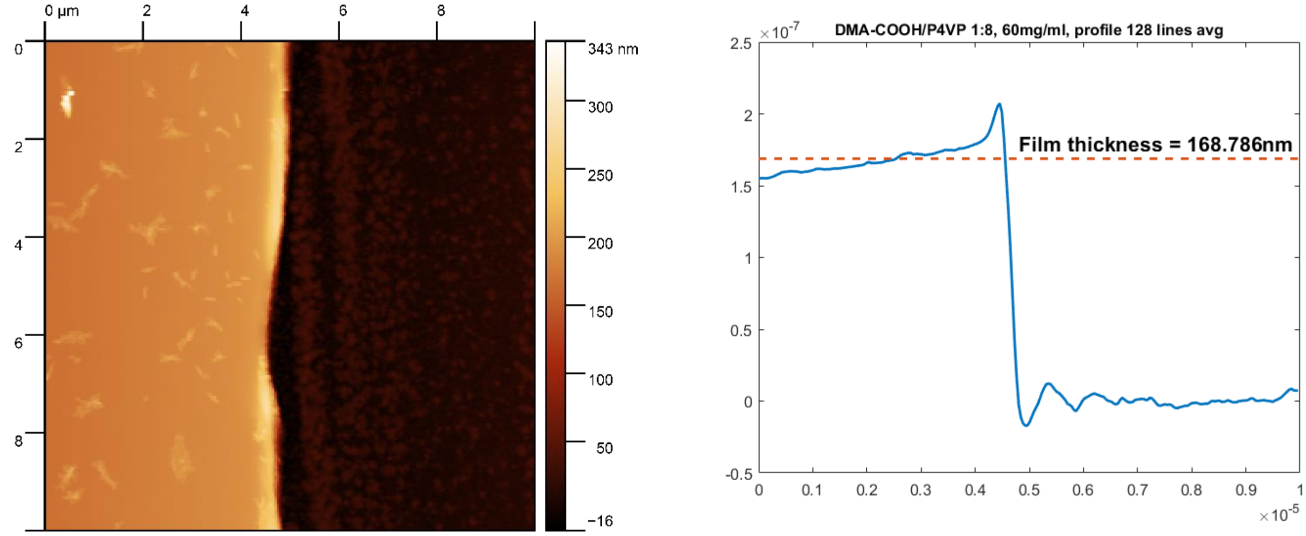

Supplementary Figure S27: AFM image of 1:8 DMA-COOH/P4VP thin film spin coated from 60 mg/ml DMF solution at 1000 rpm and the average side profile from 128 lines.

QCM measurements

The quartz crystal microbalance (QCM) experiments were done on a Q-Sense E4 instrument by Biolin Scientific, equipped with a QHM 401 humidity module. Films for QCM were directly deposited on gold coated sensors (QSX 301) by spin coating. QCM measurements were done at 23°C.

Salt solutions

The relative humidity of a closed space can be controlled by introduction of saturated salt solutions, with different salt giving different RH at given temperature.[5] Humidity control for the used QCM instrument works by having a constant flow of saturated salt solution over a membrane that allows water vapor to permeate onto the sensor surface. Because the volume between the membrane and sensor surface is very small the relative humidity equilibrates almost instantaneously after a salt solution is flowed over the membrane.

We selected a series of salts to get at wide range of humidities and decent number of data points for the determination of water absorption isotherms. Note that the selected salts should not result in less soluble salts when mixed, as briefly happens when switching from one solution to another, meaning that the no extra steps were required between changing of the solutions. One exception to this was the very first two solutions that sometimes resulted in crystals forming when mixed, most likely caused by formation of co-crystals[6]. This problem was circumvented by pumping 50 µl of milliQ water in between the two salt solutions. The salts used and their respective relative humidities at 25 °C are shown in Supplementary Table S4.

Supplementary Table S4 Salts used for QCM measurements for humidity control and their respective relative humidities.

Salt		RH [%]
Lithium Chloride	– LiCl	11.3 ± 0.27*
Magnesium Chloride	– $MgCl_2$	32.78 ± 0.16*
Magnesium Nitrate	– $Mg(NO_3)_2$	52.89 ± 0.22*
Sodium Nitrite	– $NaNO_2$	64**
Sodium Chloride	– NaCl	75.29 ± 0.12*
Potassium Chloride	– KCl	84.34 ± 0.26*
Potassium Nitrate	– KNO_3	93.58 ± 0.55*

* from ref [5]
**from ref [7]

Data analysis

QCM measurements are based on the relation between the oscillation frequency of the sensor crystal and mass of the sensor. The mass change is directly proportional to the frequency change by the Sauerbray equation[8], which can be simplified to

$$\Delta m = -\frac{C\Delta f}{n} \tag{S1}$$

where Δm is the mass change, Δf is the frequency change, C is a constant describing the sensitivity to the change of mass, and n is the overtone number ($n = 1, 3, 5, \ldots$). For quartz sensors with fundamental frequency $f_0 = 5\,\mathrm{MHz}$ the sensitivity constant is $C \approx 17.7 \cdot 10^{-9}\ \mathrm{g \cdot cm^{-2} \cdot Hz^{-1}}$. The Sauerbray equation is only valid for thin and rigid films, however. This assumption can be made when the dissipation shift $\Delta D \sim 0$ and the overtone harmonics overlap. In our experiments most samples behaved in this way, but the experiment with 1:8 CN-OH/P4VP showed significant deviation from this behaviour. This was likely caused by some error in the experiment itself rather than the sample. The data for that particular experiment was analysed with the harmonics that best adhered to the rigid behaviour. The overtone used for calculating the mass changes is indicated on each of the measured responses.

The instrumentation setup available did not offer a way to introduce dry nitrogen flow into the flow cell for 0 % RH condition. Because of this the lowest humidity condition, 11 % RH, was achieved with LiCl solution. The mass changes are therefore calculated compared to this condition. An assumption was made that at the lowest end of relative humidity the water absorption isotherm is linear and thus an approximation of the film mass at 0 % RH was made by linear extrapolation from the two lowest RH conditions, LiCl and $MgCl_2$. This gives results that quite well agree with those received from DVS experiments for the P4VP films, indicating that the assumption is agreeable.

Measured responses

These are the raw data collected for each sample. All overtones and both Δf and ΔD values are shown. The overtone used for analysis is indicated in the figure title.

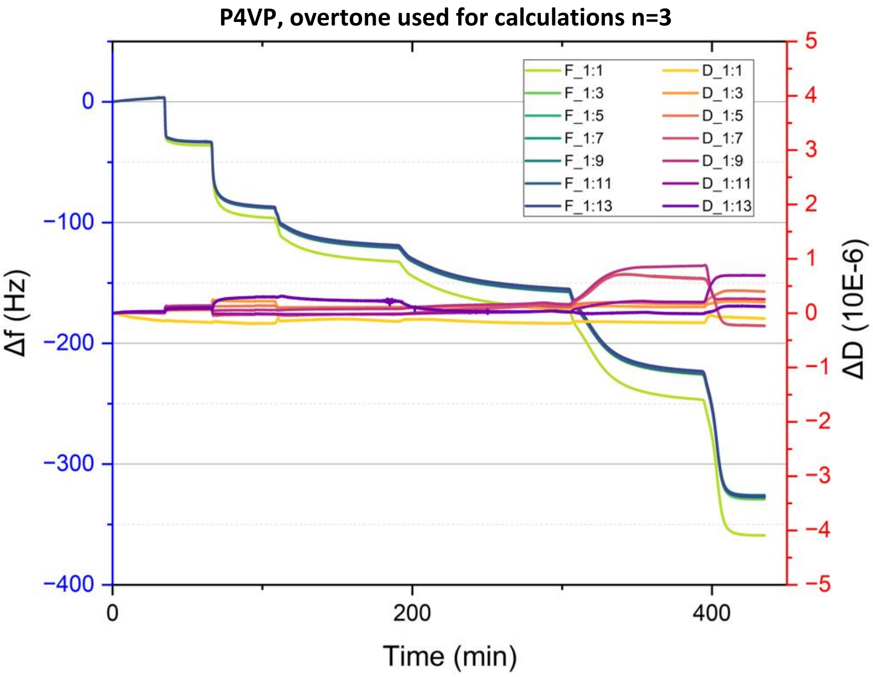

Supplementary Figure S28: QCM response of P4VP film upon changing relative humidity. Both frequency change Δf and dissipation shift ΔD are shown for all measured overtones. Each decreasing step in Δf is an increase in RH.

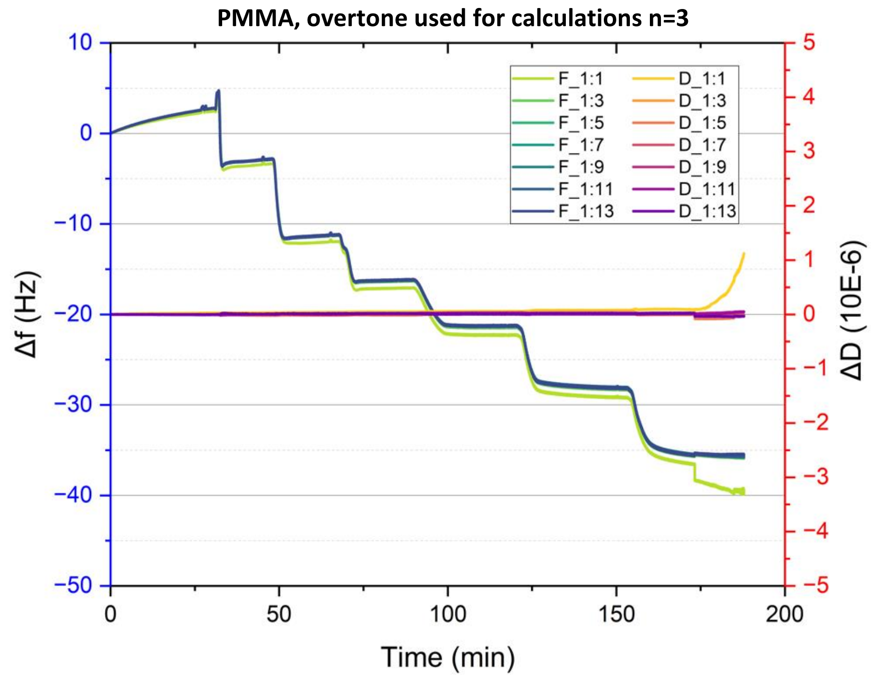

Supplementary Figure S29: QCM response of PMMA film upon changing relative humidity. Both frequency change Δf and dissipation shift ΔD are shown for all measured overtones. Each decreasing step in Δf is an increase in RH.

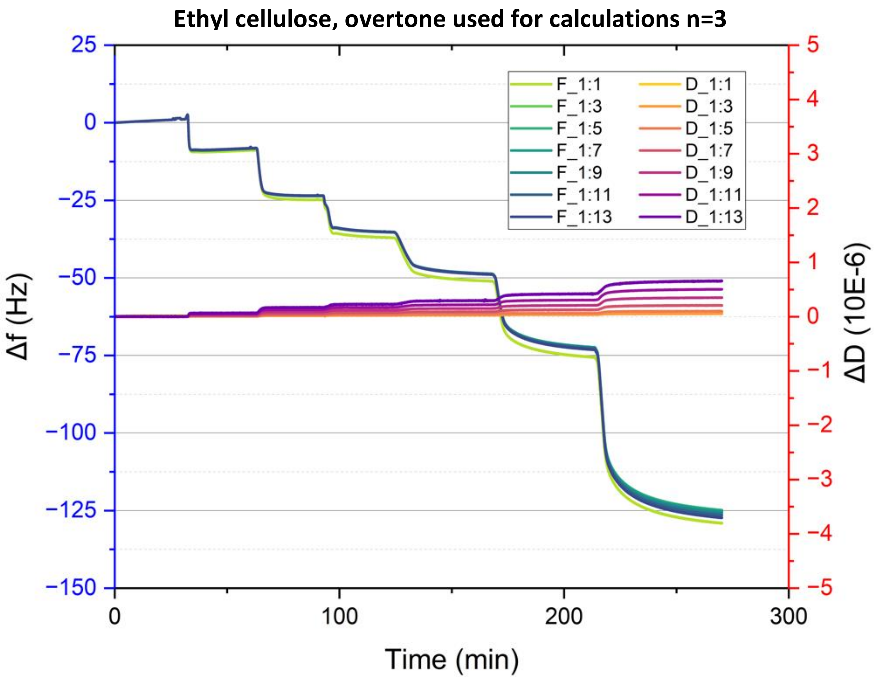

Supplementary Figure S30: QCM response of Ethyl Cellulose film upon changing relative humidity. Both frequency change Δf and dissipation shift ΔD are shown for all measured overtones. Each decreasing step in Δf is an increase in RH.

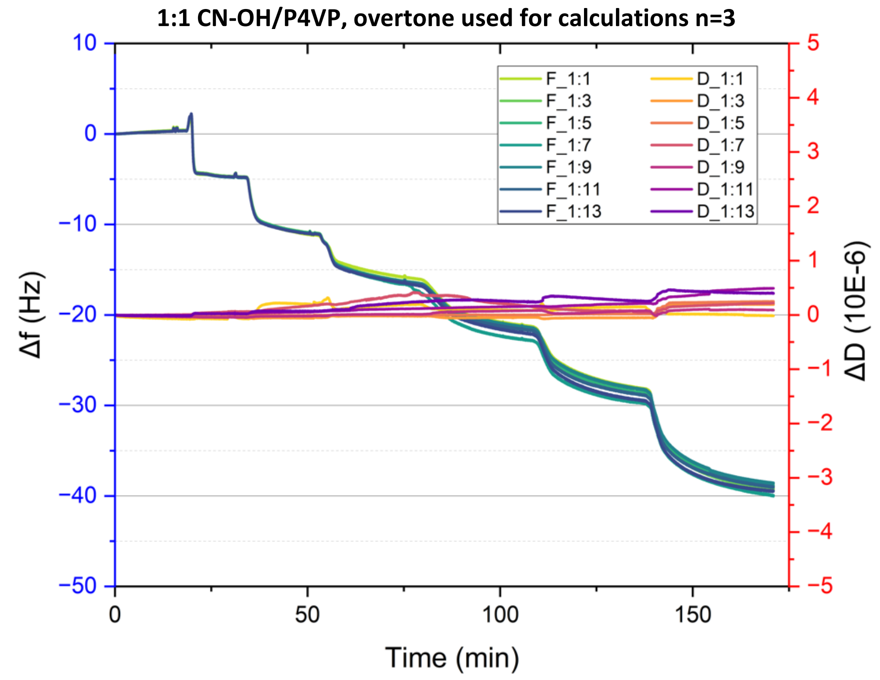

Supplementary Figure S31: QCM response of 1:1 CN-OH/P4VP film upon changing relative humidity. Both frequency change Δf and dissipation shift ΔD are shown for all measured overtones. Each decreasing step in Δf is an increase in RH.

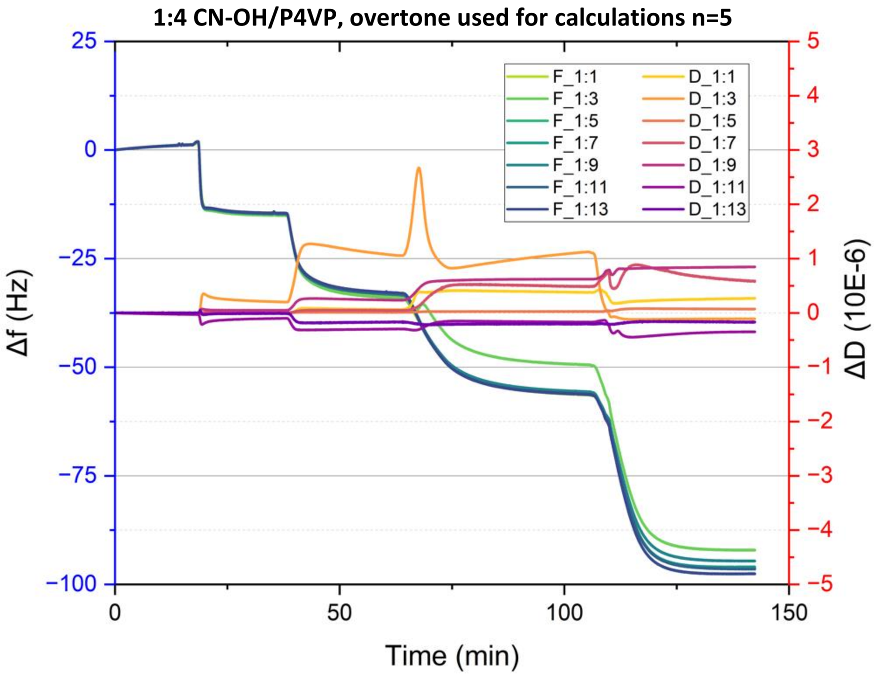

Supplementary Figure S32: QCM response of 1:4 CN-OH/P4VP film upon changing relative humidity. Both frequency change Δf and dissipation shift ΔD are shown for all measured overtones. Each decreasing step in Δf is an increase in RH.

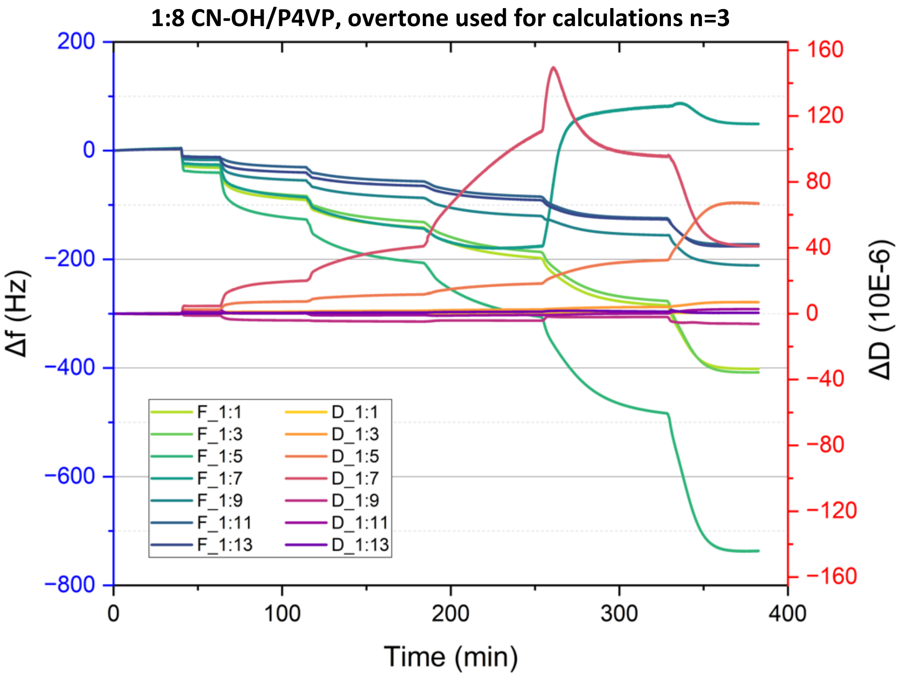

Supplementary Figure S33: QCM response of 1:8 CN-OH/P4VP film upon changing relative humidity. Both frequency change Δf and dissipation shift ΔD are shown for all measured overtones. Each decreasing step in Δf is an increase in RH.

Repeated illumination test

CN-OH/P4VP (molar ratio 1:8) film was subjected to over 1000 excitation-relaxation cycles continuously. Films were fabricated according to Supplementary Table S2. The experiment was done at 22 °C and 60 % RH. The instrumentation used was the same as described in the experimental section of the main article and the excitation cycles were automated with the function generators built into the led light sources (Lumen 1600-LED, Prior Scientific). Each excitation pulse was 600 ms long at 385 nm, followed by 60 s relaxation period. This ensured almost complete relaxation between each pulse. As a note, 600 ms pulses are rather long compared to the 100 ms that were used for most experiments, ensuring that photostationary state was reached with each pulse.

The absorption of the fully relaxed film seems to slowly reduce over the duration of the experiment, a decrease of 6,6 % upon 1032 isomerisation-relaxation cycles is observed, as shown in Supplementary Figure S34 Each excitation-relaxation cycle is included, making the data very dense, but the change in maximum absorbance is clear while the absorbance after each excitation (photo stationary state) stays rather constant. The red bars are to help visualizing the change in absorbance over time.

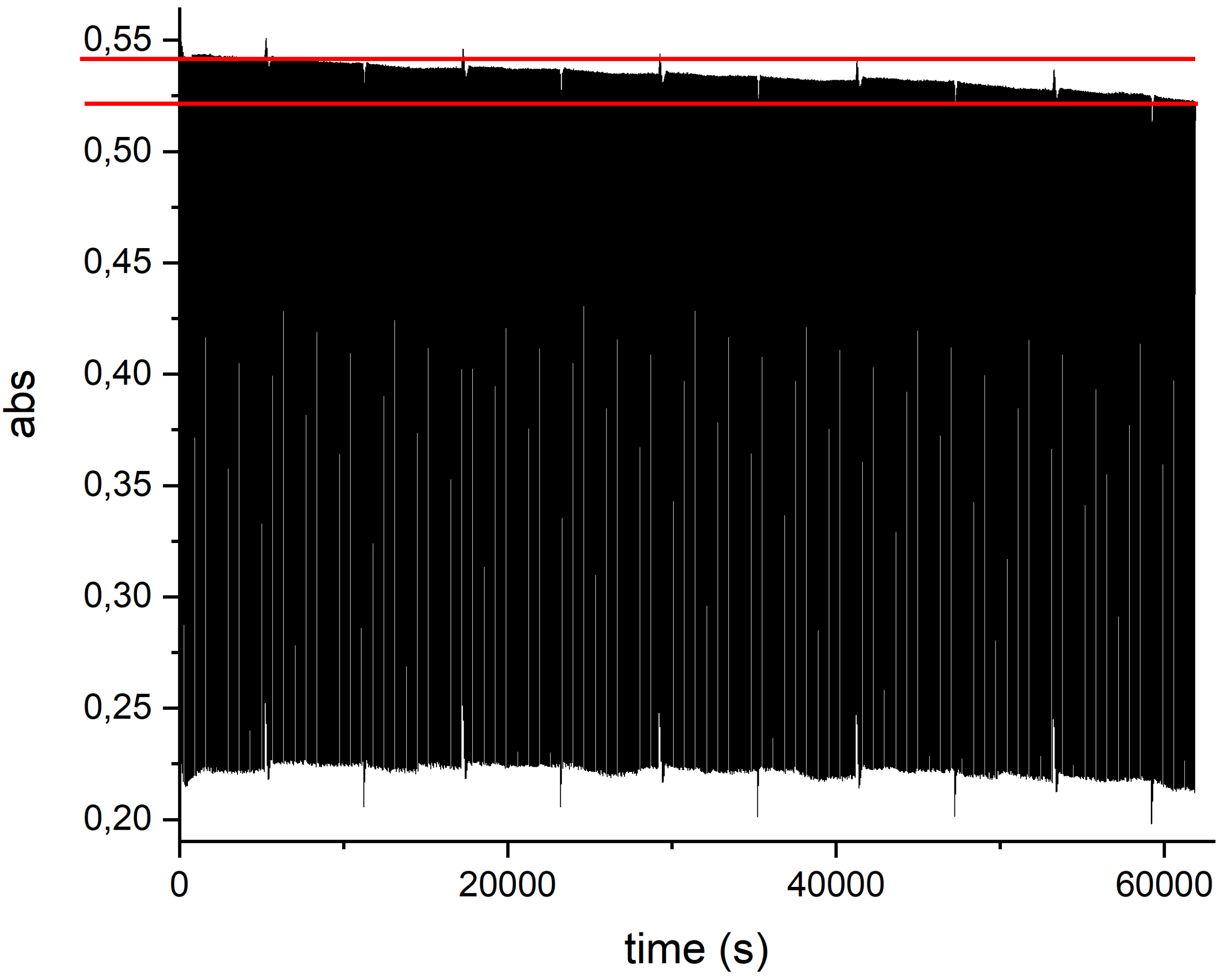

Supplementary Figure S34: Absorbance measured over 1032 isomerisation-relaxation cycles of CN-OH in P4VP. The top is absorbance of fully relaxed film, and the bottom is the photostationary state. The red bars are there to help visualizing the change in absorbance of fully relaxed film over the cycling.

Importantly, the change in maximum absorbance does not affect the kinetics of relaxation. The rate constant k for each cycle is given in Supplementary Figure S35. Notice that x-axis is the number of illumination cycles. Most of the measurements fall between $0{,}3 < k < 0{,}4\ \mathrm{s}^{-1}$ which translates to $57{,}7 < \mathrm{RH} < 59{,}8\ \%$ when calculating the relative humidity using $k_0 = 1{,}874 \cdot 10^{-4}\ \mathrm{s}^{-1}$ and $\upsilon = 0{,}1282$, as was determined for 1:8 CN-OH/P4VP films at 22 °C in the main article. The values are a bit below 60 % RH that was set for the experiment, which

can be attributed to the fact that the sensors used for the k_0 and υ characterization and the sensor used for the degradation test were fabricated in different batches and thus small differences in the film composition are possible. Also, there is no drift in k over the duration of the experiment. There are, however, several distinct datapoints way outside these bounds, both higher and lower. These are explained by the unstable humidity conditions over the experiments as the humidity controlling unit experiences some sharp fluctuations over extended periods of operation, which can be seen from the humidity data in Supplementary Figure S36. The sudden periodic increases and decreases in the humidity coincide with the increases and decreases in the measured rate constants.

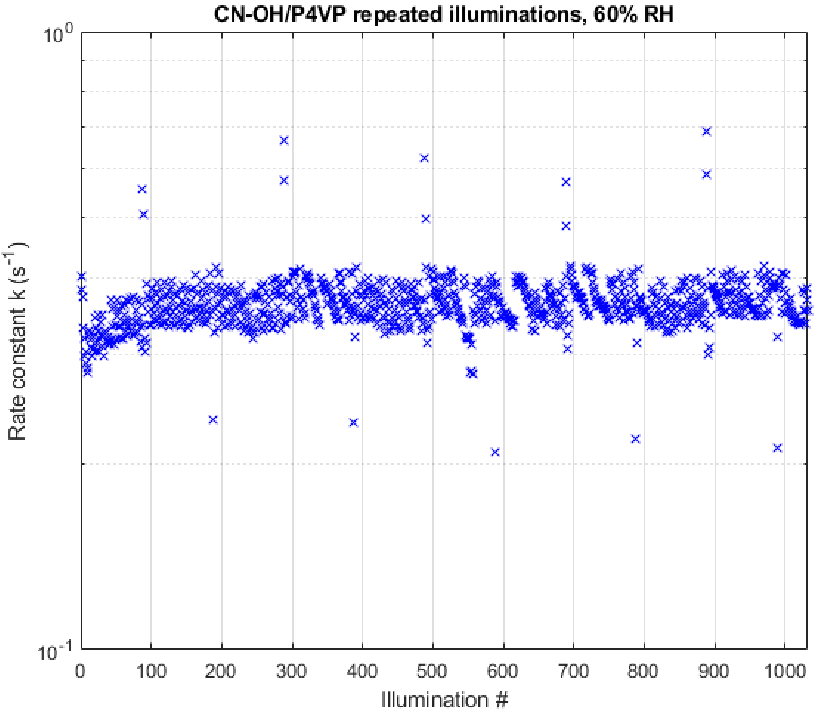

Supplementary Figure S35: Rate constant k calculated from the data shown in Figure S34 for each excitation-relaxation cycle. There is no significant drift over the course of 1000 cycles and the significantly deviating datapoints are explained by changes in humidity as shown in Figure S36.

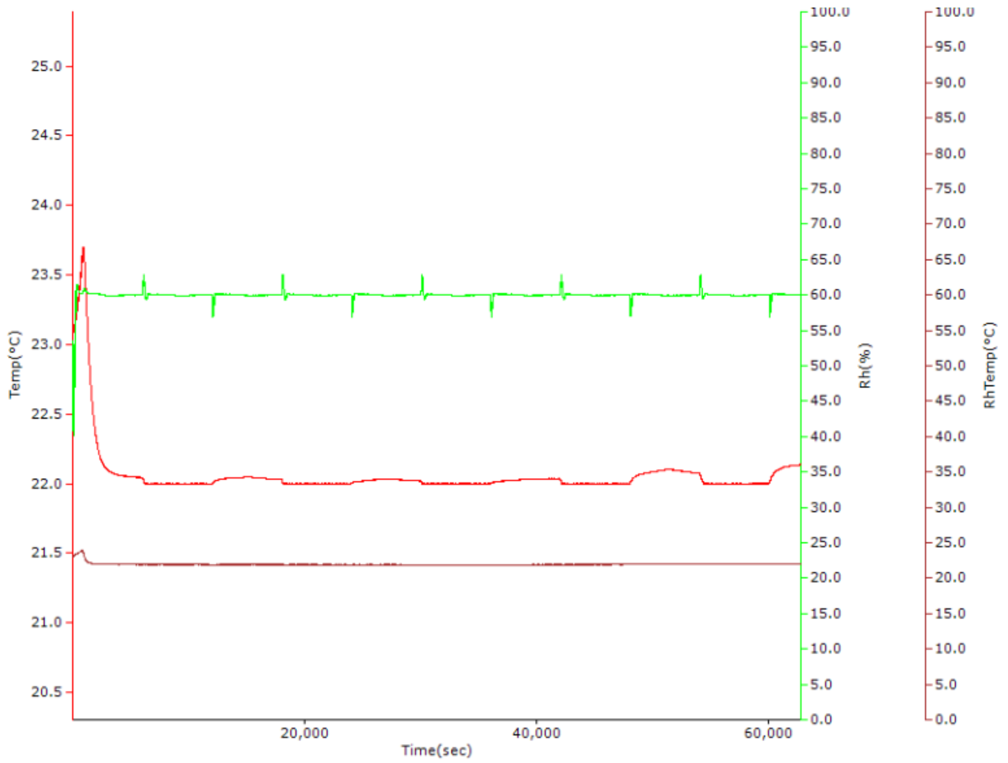

Supplementary Figure S36: Humidity and temperature data from the humidity controller over the experiment. Sharp increases and decreases in humidity can be observed periodically. These coincide with the increased and decreased rate constants measured in *Supplementary* Figure S35.

Supplementary Note 1: Absorption spectra and humidity dependency of thermal isomerisation rate of individual azobenzenes in poly (4-vinyl)pyridine

Supplementary Figures S37-S47 show the absorbance spectra and results of humidity dependency measurements for each azobenzene studied with error bars.

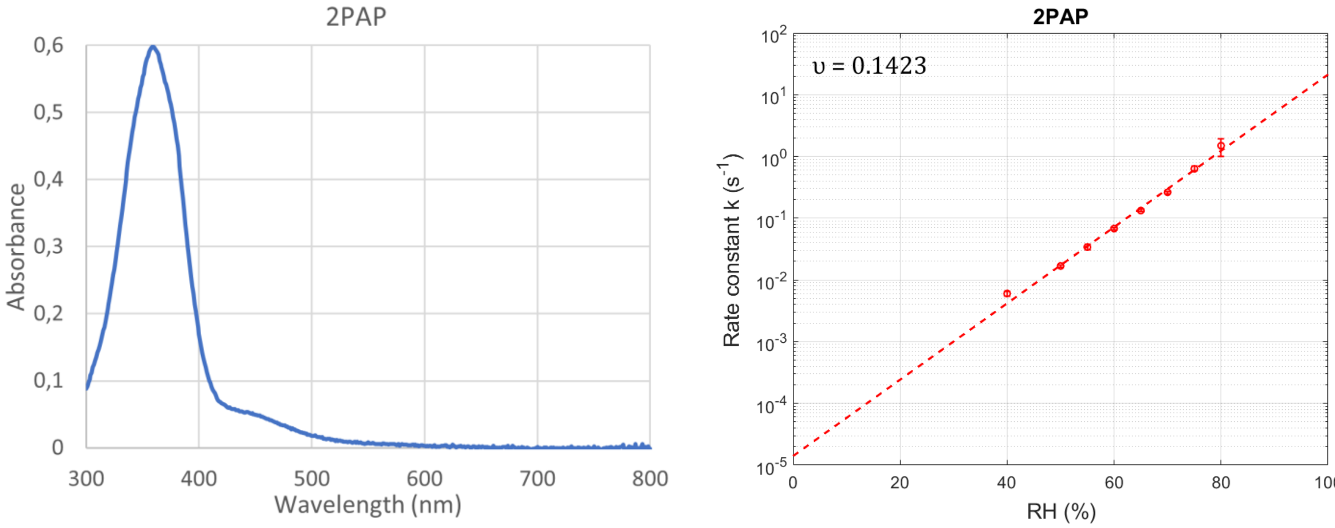

Supplementary Figure S37 Absorption spectrum and humidity dependency of thermal isomerisation rate *k*, for 2PAP, with λ describing the strength of the dependency with relation $\sim e^{\upsilon \cdot \mathrm{RH}}$. Error bars show the range of the measurements with the marker being at the mean of all measurement at the given RH.

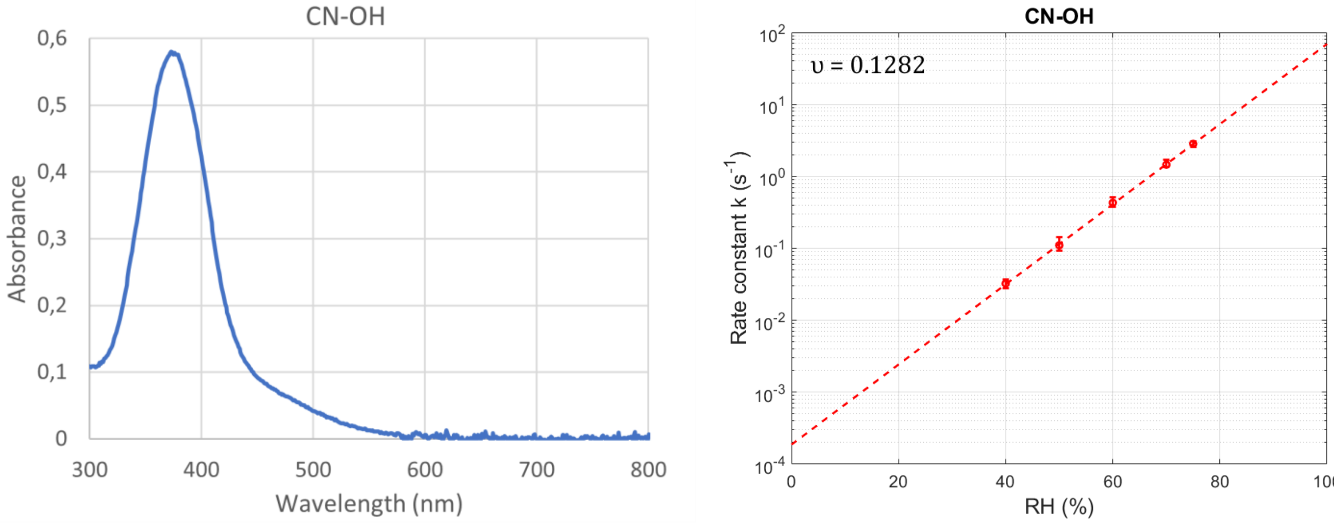

Supplementary Figure S38 Absorption spectrum and humidity dependency of thermal isomerisation rate *k*, for CN-OH, with λ describing the strength of the dependency with relation $\sim e^{\upsilon \cdot \mathrm{RH}}$. Error bars show the range of the measurements with the marker being at the mean of all measurement at the given RH.

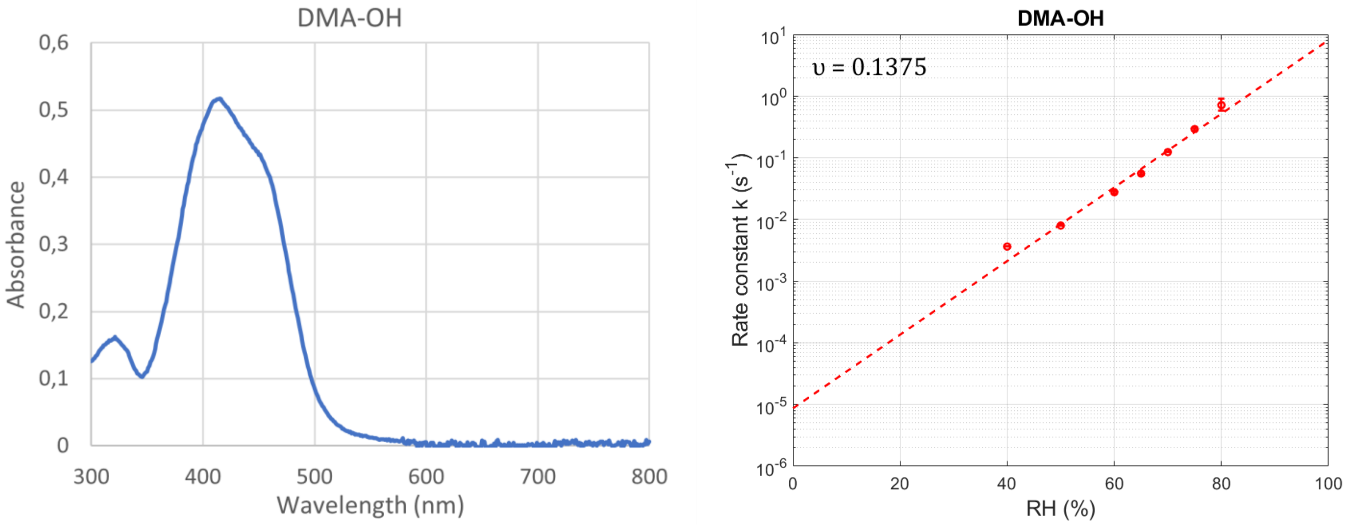

Supplementary Figure S3915 Absorption spectrum and humidity dependency of thermal isomerisation rate *k*, for DMA-OH, with λ describing the strength of the dependency with relation $\sim e^{\upsilon \cdot \mathrm{RH}}$. Error bars show the range of the measurements with the marker being at the mean of all measurement at the given RH.

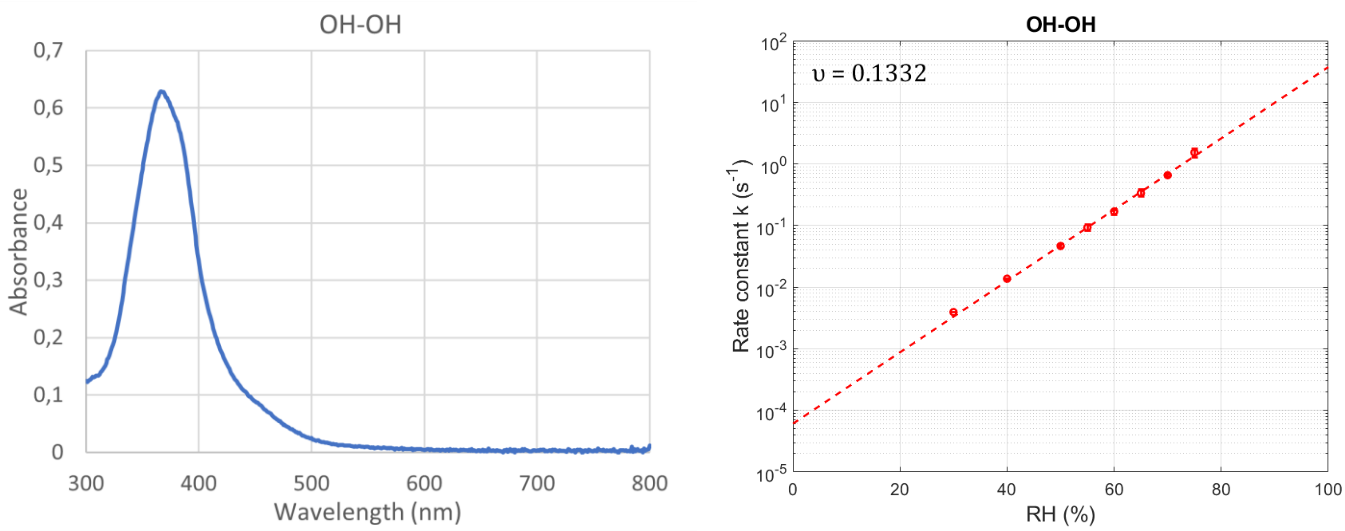

Supplementary Figure S40 Absorption spectrum and humidity dependency of thermal isomerisation rate *k*, for OH-OH, with λ describing the strength of the dependency with relation $\sim e^{\upsilon \cdot \mathrm{RH}}$. Error bars show the range of the measurements with the marker being at the mean of all measurement at the given RH.

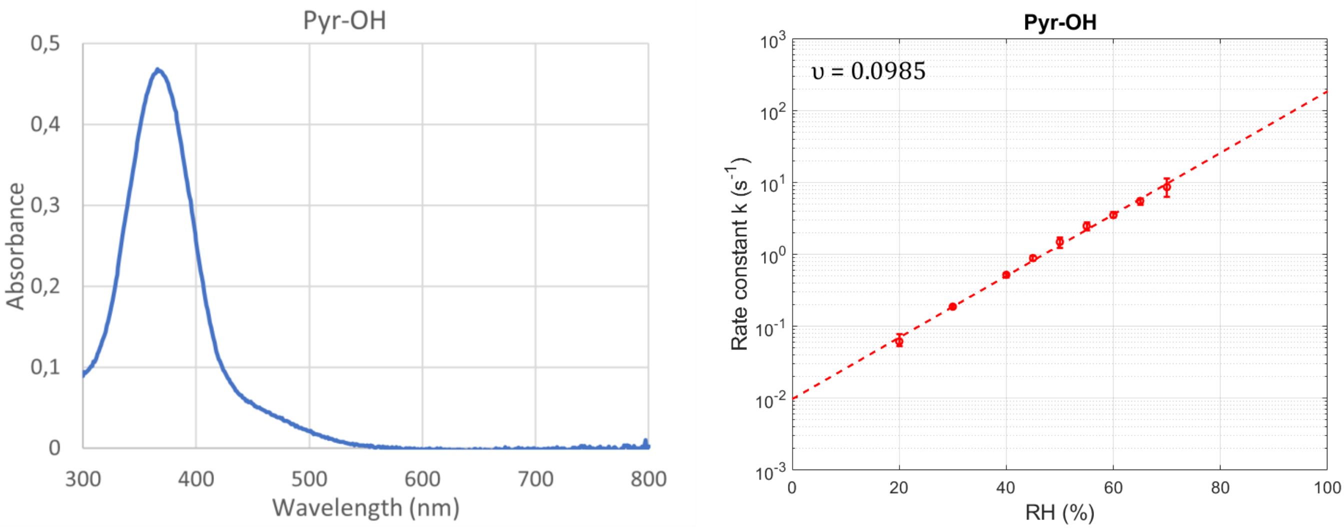

Supplementary Figure S41 Absorption spectrum and humidity dependency of thermal isomerisation rate *k*, for Pyr-OH, with λ describing the strength of the dependency with relation $\sim e^{\upsilon \cdot \mathrm{RH}}$. Error bars show the range of the measurements with the marker being at the mean of all measurement at the given RH.

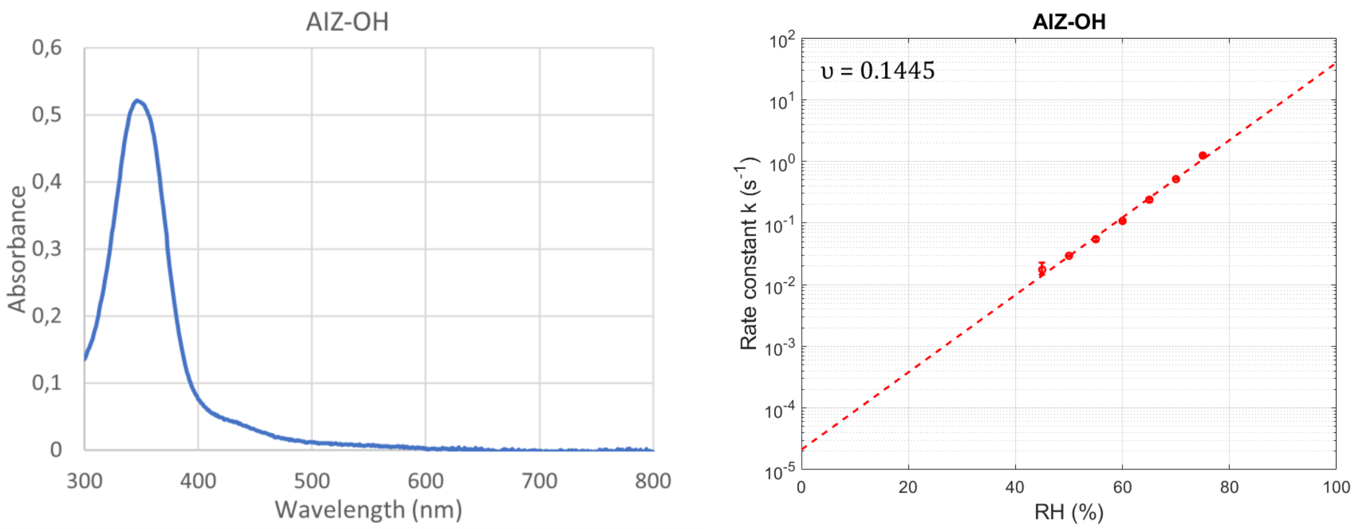

Supplementary Figure S42 Absorption spectrum and humidity dependency of thermal isomerisation rate *k*, for AIZ-OH, with λ describing the strength of the dependency with relation $\sim e^{\upsilon \cdot \mathrm{RH}}$ Error bars show the range of the measurements with the marker being at the mean of all measurement at the given RH.

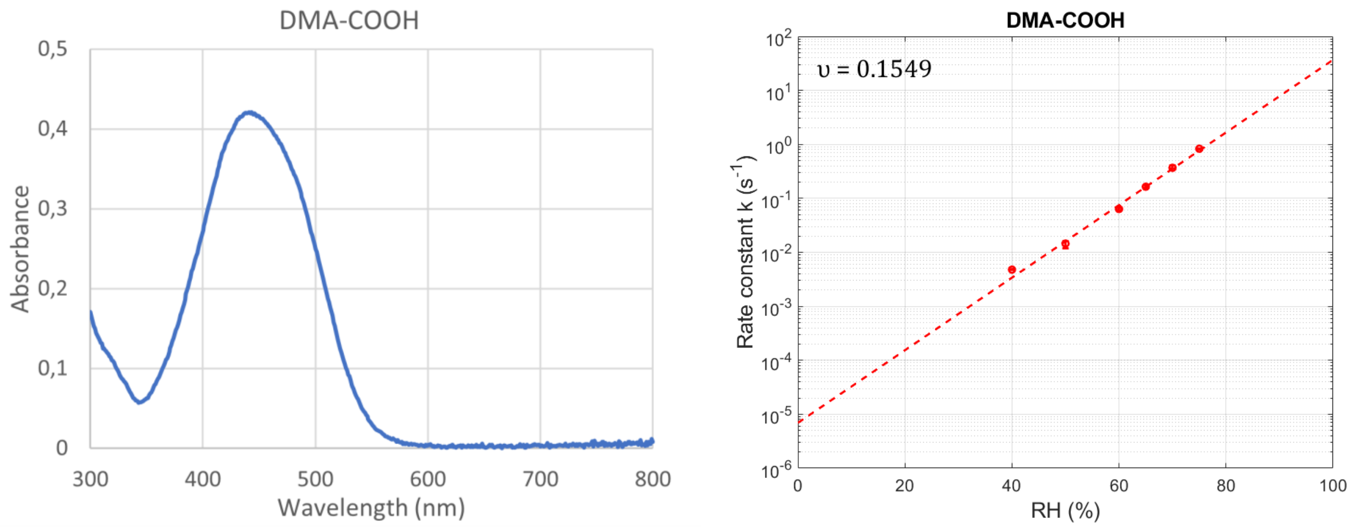

Supplementary Figure S43 Absorption spectrum and humidity dependency of thermal isomerisation rate *k*, for DMA-COOH, with λ describing the strength of the dependency with relation $\sim e^{\upsilon \cdot \mathrm{RH}}$. Error bars show the range of the measurements with the marker being at the mean of all measurement at the given RH.

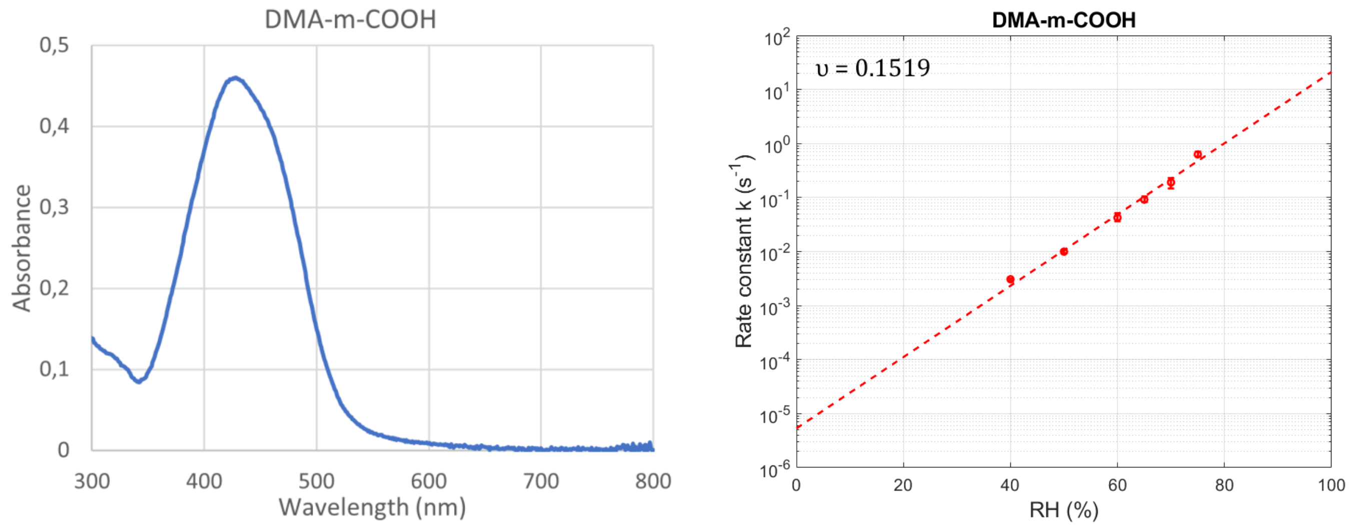

Supplementary Figure S44 Absorption spectrum and humidity dependency of thermal isomerisation rate *k*, for DMA-m-COOH, with λ describing the strength of the dependency with relation $\sim e^{\upsilon \cdot \mathrm{RH}}$. Error bars show the range of the measurements with the marker being at the mean of all measurement at the given RH.

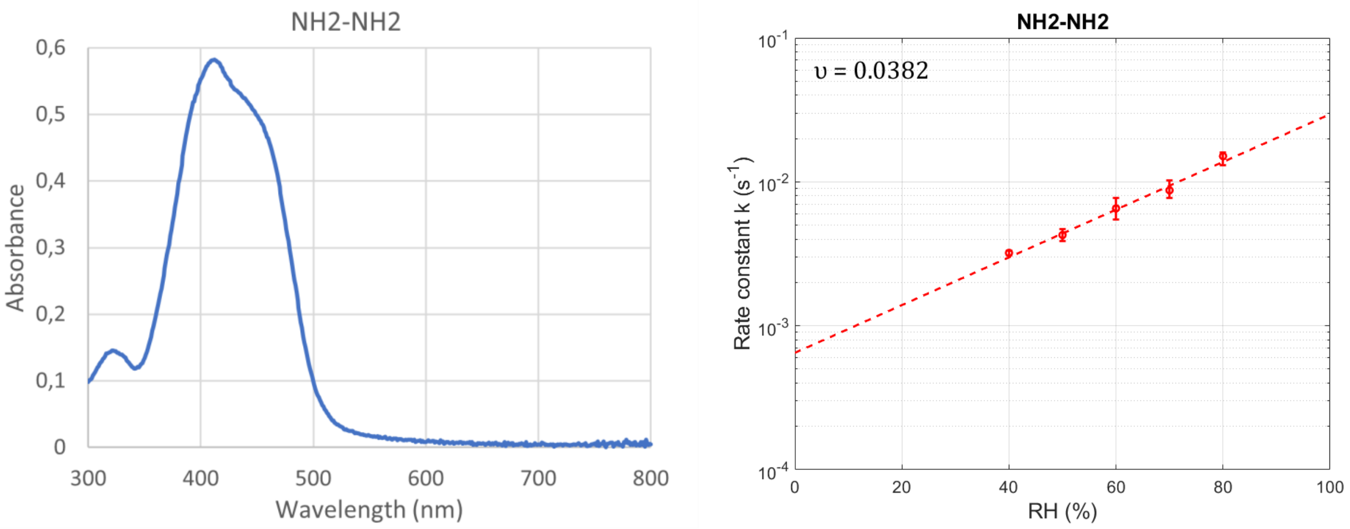

Supplementary Figure S45 Absorption spectrum and humidity dependency of thermal isomerisation rate *k*, for NH_2-NH_2, with λ describing the strength of the dependency with relation $\sim e^{\upsilon \cdot \mathrm{RH}}$. Error bars show the range of the measurements with the marker being at the mean of all measurement at the given RH.

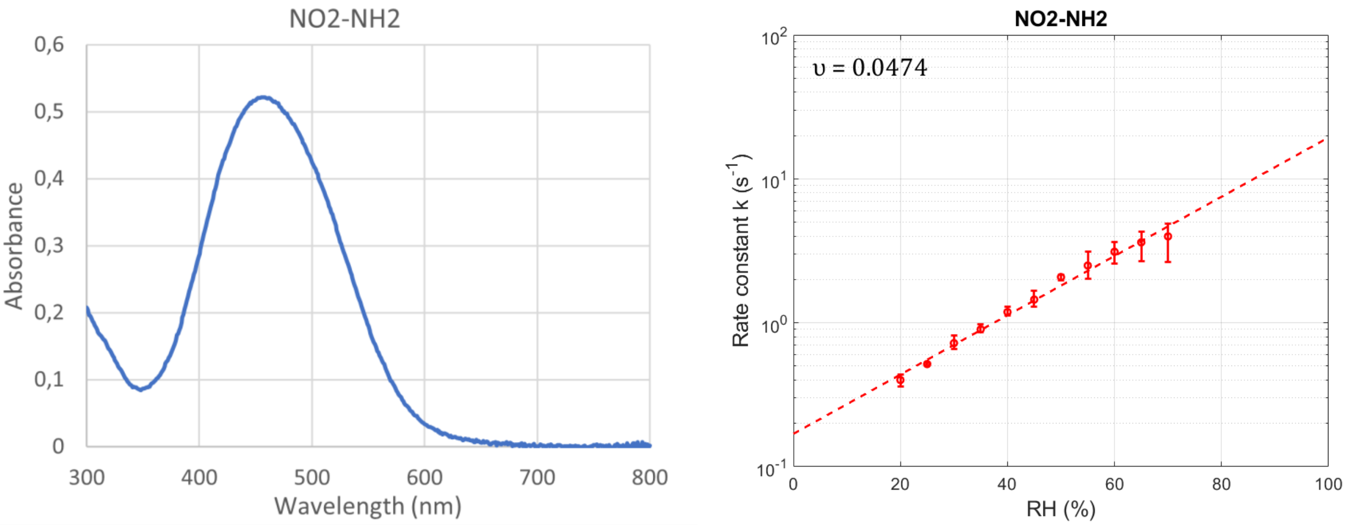

Supplementary Figure S46 Absorption spectrum and humidity dependency of thermal isomerisation rate *k*, for NO_2-NH_2, with λ describing the strength of the dependency with relation $\sim e^{\upsilon \cdot \mathrm{RH}}$. Error bars show the range of the measurements with the marker being at the mean of all measurement at the given RH.

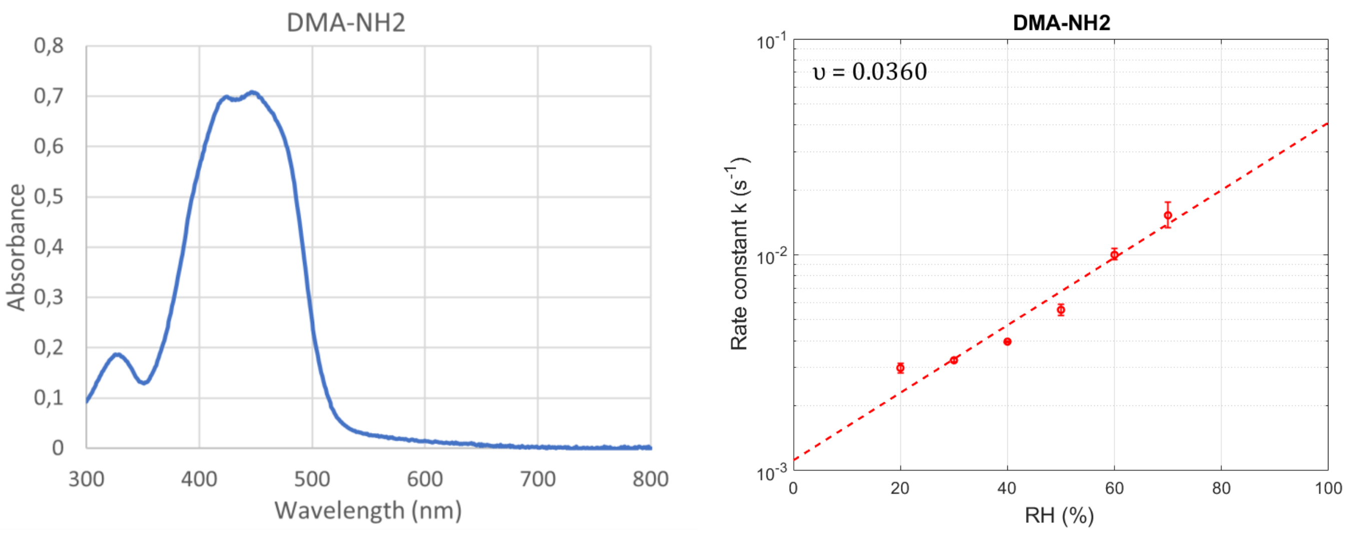

Supplementary Figure S47 Absorption spectrum and humidity dependency of thermal isomerisation rate *k*, for DMA-NH_2, with λ describing the strength of the dependency with relation $\sim e^{\upsilon \cdot \mathrm{RH}}$. Error bars show the range of the measurements with the marker being at the mean of all measurement at the given RH.

Supplementary References

1. Kortekaas, L., Simke, J., Kurka, D. W. & Ravoo, B. J. Rapid Photoswitching of Low Molecular Weight Arylazoisoxazole Adhesives. *ACS Appl. Mater. Interfaces* **12**, 32054–32060 (2020).
2. Basu Baul, T. S., Dutta, D., Duthie, A. & Guedes Da Silva, M. F. C. Perceptive variation of carboxylate ligand and probing the influence of substitution pattern on the structure of mono- and di-butylstannoxane complexes. *Inorganica Chim. Acta* **455**, 627–637 (2017).
3. Lu, H., Wang, J. & Song, X. Supramolecular Liquid Crystals Induced by Intermolecular Hydrogen Bonding. *Mol. Cryst. Liq. Cryst.* **537**, 93–102 (2011).
4. Suwanprasop, S., Suksorn, S., Nhujak, T., Roengsumran, S. & Petsom, A. Petroleum Markers Synthesized from *n* -Alkylbenzene and Aniline Derivatives. *Ind. Eng. Chem. Res.* **42**, 5054–5059 (2003).
5. Greenspan, L. Humidity fixed points of binary saturated aqueous solutions. *J. Res. Natl. Bur. Stand. Sect. Phys. Chem.* **81A**, 89 (1977).
6. Cai, H.-Q., Li, H.-J., Wang, M., Wang, C.-C. & Yi, H.-B. Microscopic insight into the ion aggregation characteristics in aqueous MgCl2 and MgCl2–LiCl solutions: Implications for Mg2+/Li+ separation. *J. Mol. Liq.* **273**, 374–382 (2019).
7. The Engineering ToolBox (2014). *Saturated Salt Solutions - Controlling Air Humidity.* [online] Available at: https://www.engineeringtoolbox.com/salt-humidity-d_1887.html [Accessed 20.03.2024].
8. Sauerbrey, G. Verwendung von Schwingquarzen zur Wägung dünner Schichten und zur Mikrowägung. *Z. Für Phys.* **155**, 206–222 (1959).
